# Seed Germination Behavior, Growth, Physiology and Antioxidant Metabolism of Four Contrasting Cultivars under Combined Drought and Salinity in Soybean

**DOI:** 10.3390/antiox11030498

**Published:** 2022-03-03

**Authors:** Naheeda Begum, Mirza Hasanuzzaman, Yawei Li, Kashif Akhtar, Chunting Zhang, Tuanjie Zhao

**Affiliations:** 1 National Center for Soybean Improvement, Key Laboratory of Biology and Genetics and Breeding for Soybean, Ministry of Agriculture, State Key Laboratory for Crop Genetics and Germplasm Enhancement, Nanjing Agricultural University, Nanjing 210095, China; t2020106@njau.edu.cn (N.B.); 2018201083@njau.edu.cn (Y.L.); 2018801229@njau.edu.cn (C.Z.); 2 Department of Agronomy, Faculty of Agriculture, Sher-e-Bangla Agricultural University, Dhaka 1207, Bangladesh; mirzahasanuzzaman@sau.edu.bd; 3 State Key Laboratory for Conservation and Utilization of Subtropical Agro-Bio-Resources, College of Life Science and Technology, Guangxi University, Nanning 530004, China; kashif@zju.edu.cn

**Keywords:** *Glycine max*, seed germination, photosynthesis, antioxidant enzymes, secondary metabolites, drought, salinity

## Abstract

Drought and salinity stresses are persistent threat to field crops and are frequently mentioned as major constraints on worldwide agricultural productivity. Moreover, their severity and frequency are predicted to rise in the near future. Therefore, in the present study we investigated the mechanisms underlying plant responses to drought (5, 10 and 15% polyethylene glycol, PEG-6000), salinity (50, 100, and 150 mM NaCl), and their combination, particularly at the seed germination stage, in terms of photosynthesis and antioxidant activity, in four soybean cultivars, viz., PI408105A (PI5A), PI567731 (PI31), PI567690 (PI90), and PI416937 (PI37). Results showed that seed germination was enhanced by 10% PEG and decreased by 15% PEG treatments compared to the control, while seed germination was drastically decreased under all levels of NaCl treatment. Furthermore, combined drought and salinity treatment reduced plant height and root length, shoot and root total weights, and relative water content compared with that of control. However, the reductions were not similar among the varieties, and definite growth retardations were observed in cultivar PI5A under drought and in PI37 under salinity. In addition, all treatments resulted in substantially reduced contents of chlorophyll pigment, anthocyanin, and chlorophyll fluorescence; and increased lipid peroxidation, electrolyte leakage, and non-photochemical quenching in all varieties of soybean as compared to the control plants. However, proline, amino acids, sugars, and secondary metabolites were increased with the drought and salinity stresses alone. Moreover, the reactive oxygen species accumulation was accompanied by improved enzymatic antioxidant activity, such as that of superoxide dismutase, peroxidase, catalase, and ascorbate peroxidase. However, the enhancement was most noticeable in PI31 and PI90 under both treatments. In conclusion, the cultivar PI31 has efficient drought and salinity stress tolerance mechanisms, as illustrated by its superior photosynthesis, osmolyte accumulation, antioxidative enzyme activity, and secondary metabolite regulation, compared to the other cultivars, when stressed.

## 1. Introduction

Abiotic stress is a serious environmental issue that restricts agricultural production, and therefore, global climate change is of great relevance [1]. The most common abiotic stressors that adversely affect plant development and growth are drought and soil salinity [2]. It has been noted that plants in around 10% and 40% of the world’s arable land suffer from soil salinity and drought stress [3]. Drought stress inhibits the performance of essential biochemical mechanisms, such as the photosynthetic process [4], nutritional absorption, and osmotic adjustments by influencing the functions of essential enzymes [5]. Drought reduces the water potency in cells, resulting in plant water loss and wilting. Water deficiency for an extended period disrupts membrane integrity, removing the membrane’s selective permeability and causing other severe damage. In plants, photosynthesis is one of the most critical metabolic processes that regulate photosynthetic efficiency, thereby regulating plant growth and development. Drought and salt stress have significant impacts on photosynthetic performance, causing several biochemical and physiological changes in plants [3,6,7]. Therefore, detecting the changes in photosynthesis to environmental factors, especially in drought and salinity conditions, is helpful for facilitating plants to adjust to environmental stresses, such as extreme salt and lack of water [3].

Similarly, salinity is another critical environmental stressor that due to toxicity has a negative impact on plants’ productivity globally [8]. Accumulation of Na^+^ in plant tissues, along with the proliferation of various other toxic ions, causes biological imbalances in plant cells, which in turn result in desperate destruction to the structural and functional permanence of plants’ photosynthesis [5]. The processes through which salinity stress affects plant photosynthetic activity include: triggering the over-excitation of photosystem II, which tends to result in more non-radioactive energy loss [9]; lowering the apparent quantum efficiency of photosynthetic activity [9,10]; accumulating reactive oxygen species (ROS), resulting in oxidative damage, which disrupts cellular homeostasis and decreases photosynthetic efficiency and plant growth [11]. Plants, in general, have established natural morpho-physiological protective mechanisms to conquer stress conditions [12], the most significant of which is the antioxidant defense system, which plays an important role in scavenging overproduced ROS [13]. To counteract the oxidative damage induced by ROS, plants normally upregulate antioxidative defense and antioxidant enzymes [14]. Previous research revealed that enzymatic antioxidants play important roles in stress resistance and respond as a defense mechanism in plants [15]. Hence, plants with a stronger antioxidant defense system may be more stress resistant.

In addition, previously, research has focused on elucidating the mechanisms underlying drought and salt stress in plants, and screening and producing stress-resistant cultivars. The soybean (*Glycine max* (L.) Merr), as a key commercial crop, is an important legume widely adopted and cultivated worldwide for its protein content, oil (for consumption), and fatty acids. However, several abiotic variables, such as temperature, flooding, drought, salt, and acidity, significantly affect soybean crop’s overall efficiency [16]. Consequently, crop yields must be protected from increasing and more frequent abiotic stresses, particularly drought and salinity, in current and future climates. For soybean crops, drought stress mainly occurs during the growing season, leading to considerable yield loss and quality deterioration, mainly in arid and semi-arid zones. Drought stress also disturbs carbon assimilation and plant phenology in the soybean [17]. Therefore, in order to fulfill future food demands, breeding efforts will need to improve not only current yields but also salinity and drought resistance.

To counteract the detrimental effects of stress on soybeans, a variety of approaches have been established and implemented, the most common of which include agricultural practices and genetic improvements to soybean cultivars [18]. Rainwater and irrigation water are also being used increasingly; although irrigation system adaption is region-specific and significantly increases soybean production costs [19]. Therefore, the most economic way of sustainable soybean cultivation is to develop soybean genotypes having the ability to germinate and survive under severe stress conditions [20,21]. Evidently, enhancing soybean cultivars’ stress tolerance is critical for protecting yield gains [22]. Previously the PI 416937 soybean has been shown to have a substantially wider root system than other lines, which could be one component in improving drought resistance. Another investigation recently discovered that PI 416937 is implicated in soybean resistance to aluminum (Al) stress [23]. In addition, two soybean germplasms, PI 567690 and PI 567731, were recently found to consistently express drought tolerance in the field compared to two drought-sensitive genotypes [24]. Although these studies evaluated that these soybean genotypes show drought stress tolerance, the mechanisms underlying the seed germination traits and early seedling growth of these PIs under drought, salinity, and combined stresses require further investigation.

Drought and high salinity conditions are becoming more severe and frequent in agricultural areas, hampering plant growth and development and resulting in significant crop loss. Therefore, identifying the stress tolerant soybean cultivars and significant regulators involved in organized responses to concurrent stresses will be useful. The soybean is an important factor of global food security for both human beings and animals, and a significant means of supplying edible protein and cooking oil. The food demand has increased as the world’s population has grown, but agricultural land has reduced. As a result, soybean cultivation will be critical in utilizing arid soil. Soybean performance under combined salinity and drought stress has received less attention to date, even though understanding the combined salinity and drought stress tolerance mechanisms and improving resistance are critical for soybean production.

Therefore, the main objective of the present study was to evaluate physiological and biochemical modulations in four contrasting soybean genotypes concerning germination and early seedling growth under drought, high salinity, and both. Physiological and biochemical analyses were used to investigate the mechanisms of drought and salinity stress tolerance in contrasting soybean genotypes. In particular, we showed that antioxidant enzymes’ activities, osmolyte accumulation, and secondary metabolite regulation all have significant roles in salinity and drought stress responses. The present observations could serve as a conceptual framework for selecting and breeding drought and salinity-resistant soybean cultivars.

## 2. Materials and Methods

### 2.1. Experimental Plant Materials

This study used genetically diverse soybean genotypes, PI567731 (PI31), PI416937 (PI37) PI567690 (PI90), and PI408105A (PI5A). Accession numbers PI90, PI31, PI37, and PI5A were selected as contrasting genotypes with drought tolerance [25,26], aluminum resistance [23,27], and flooding tolerance. Other information about soybean cultivars that were used in the present study is provided in Table 1. The respective soybean cultivars seeds were allowed to dry naturally before being cleaned and placed in a tightly sealed glass container, which was then kept at 4 °C in the refrigerator until needed for experiments.

### 2.2. Seeds’ Germination Treatment

Mature seeds were sterilized for 5 min in a sodium hypochloride solution before being rinsed with distilled water. For each cultivar, 30 healthy seeds were placed in Petri dishes (9 cm length diameter) with two layers of Whatman no. 1 filter paper with six different treatments as follows: (i) distilled water (control, CK), (ii) 5% polyethylene glycol-6000, PEG, (iii) 10% PEG, (iv) 15% PEG, (v) 50 mM NaCl, and (vi) 100 mM NaCl and (vii) 150 mM NaCl respectively. Germination and growth analyses of four different available soybean cultivars (PI31, PI90, PI37 and PI5A) were conducted in a growth chamber, with a 16 h light/8 h dark day/night pattern at temperatures of 25 °C. There were three replications for each cultivar. Germinated seeds were removed after counting to avoid inaccuracies in germination records.

### 2.3. Seedlings’ Treatments

Seeds of four soybean materials were sown in the growth chamber under the same conditions described in the previous paragraph. Seedlings were transplanted into pots filled with a Vermiculite, peat (2:1: *w*/*w*) mixture when they were 5 days old. The pots were irrigated with half-strength Hoagland’s nutrient solution every other day. After 20 days of growth with regular management, seedlings were exposed to one of four treatments—viz., (1) control, (2) 15% PEG-6000, (3) 150 mM NaCl, or (4) 15% PEG-6000 + 150 mM NaCl (Table S1). The study used a completely randomized design with four factors (genotypes, drought, salinity, and combined stress), four genotypes (PI31, PI90, PI37, and PI5A), and four treatments (control; drought, 15% PEG-6000; salinity, 150 Mm NaCl; and combined stress, 15% PEG-6000 + 150 mM NaCl) (Table S1). Salt stress was applied by adding NaCl to the Hoagland solution to achieve a final concentration of 150 mM NaCl. Drought stress was addressed by dissolving 15% PEG in half-strength Hoagland’s nutrient solution. For combined treatments, 15% PEG (150 mM combined) was added to the Hoagland solution, and the plants were irrigated with the Hoagland solution. The leaf samples were collected from each of the four treatments after the seedlings had been treated for seven days. After that, the samples were immediately frozen with liquid nitrogen and maintained in the fridge until measurements of chlorophyll (Chl) pigment, anthocyanin, Chl fluorescence, lipid peroxidation, electrolyte leakage, osmolytes, antioxidative enzymes’ activity levels, and soluble protein content could be performed.

### 2.4. Seed Germination Calculation

Seed germination was tracked daily during the first five days of the experiment. When the radicle length was more than 2 mm, seeds were considered germinated [28]. The number of germinated seeds by the last day of the total number of seeds was used for the germination percentage (GP) [29]. Germination energy (GE) was calculated by using the formula below:

(1) $$\left(\mathrm{GE},\;\%\right)=\frac{\mathrm{No}.\;\mathrm{of}\;\mathrm{germinated}\;\mathrm{seeds}\;\mathrm{within}\;3\;\mathrm{days}}{\mathrm{total}\;\mathrm{no}.\;\mathrm{of}\;\mathrm{seeds}\;\mathrm{planted}}\times 100$$

The Germination index (GI) was computed using Equation,

(2) $$\mathrm{Germination}\;\mathrm{index}\;\left(\mathrm{GI}\right)=\frac{\sum \mathrm{Gt}}{\mathrm{Dt}}\;$$

In the formula, number of seeds germinated on a given day is represented by Gt, and Dt represents the number of days from the start of the experiment.

### 2.5. Fresh and Dry Biomass, Plant Height, and Relative Water Content (RWC) Measurements

After harvesting, the plant height was measured with a measuring tape. After that, the seedlings were divided into two groups: shoots and roots. The fresh weight (FW) of the shoots and that of the roots were calculated using an electronic weight balance, and the dry masses of the shoot and roots were determined after oven drying at 60 °C for 72 h.

The RWC was determined using the WEATHERLEY [30] method. The 8 mm diameter leaf discs were weighed and immersed for 4 h in deionized water, following which the turgid weight was recorded (TW). After the discs had been dried in the oven, the dry biomass (DW) was estimated. For calculation, we utilized the formula below.

(3) $$\mathrm{RWC}\left(\%\right)=\frac{\left(\mathrm{FW}-\mathrm{DW}\right)}{\left(\mathrm{TW}-\mathrm{DW}\right)}\times 100$$

### 2.6. Chlorophyll Fluorescence, Photosynthetic Pigments, and Anthocyanin Content

The plants’ uppermost completely grown, fresh, and healthy leaves were selected after 7 days of stress treatments to assess Chl fluorescence. To ensure that all maximum yield of photosystem II (PSII, Fv/Fm) were dark-adapted, the leaves were dark-acclimated for at least 30 min prior to collecting these measurements. Then, a beam of saturating red light triggered the Chl fluorescence fluorometer transients (MINI-Imaging-PAM, (HeinZWalZ GmbH Fffeltrich, Germany). In addition, fresh leaves were homogenized in acetone at a concentration of 80%. A spectrophotometer was used to determine the contents of Chl a, Chl b, and carotenoids at 645, 663, and 480 nm, respectively, according to [31].

For determining anthocyanin content, approximately 500 mg of the leaf sample was taken in a 5 mL methanol solution, 6 M HCL, and drained at 4 °C for 24 h in an airtight container. The extract was then mixed with 1 mL distilled water and 2 mL chloroform in a 2 mL aliquot. A spectrophotometer was used to evaluate the absorbance of the upper chloroform layer containing extracted anthocyanins at 530 nm, by following [32]

### 2.7. Determination of Proline, Sugars, and Free Amino Acid and Soluble Protein

The proline activity was calculated according to the protocols established by Bates et al. [33]. The plant tissues were homogenized with 3% sulphosalicylic acid and centrifuged for 10 min at 3600 rpm before being tested. The 2 mL solution was mixed with ninhydrin and glacial acetic acid before being incubated at 100 °C for 1 h before being discarded, after which the optical density of the solution was measured at 520 nm. The sugar content in leaves was determined by mixing 0.1 g dried ground samples in 80 percent ethanol and measuring the extract absorbance at 620 nm, as described by Fong et al. [34]. Furthermore, the contents of amino acids were assayed by following the methods of Sadasivam and Manickam [35].

Fresh leaf tissue (0.5 g) was pulverized in a 1 mL extract solution that contained 1 mM ascorbic acid, 1 mM KCl, 0.5M KP buffer (pH 7.0), mercaptoethanol, and glycerol in an ice-cold mortar and pestle. The mixture was centrifuged for 15 min at 11,500× *g*. The supernatant was used to assess enzyme activity as a soluble protein solution. Each sample’s protein concentration was measured using the [36] method.

### 2.8. Lipid Peroxidation and Electrolyte Leakage (EL)

According to the protocols developed by Heath and Packer [37], the lipid peroxidation content was determined. To obtain the final product, fresh tissues were extracted with 1 percent trichloroacetic acid and centrifuged at 10,000× *g* for 5 min. The supernatant was then heated to 95 °C for 30 min before being diluted with 0.5% thiobarbituric acid. After cooling, the samples were centrifuged at 5000× *g* for 15 min to measure absorbance at 532 and 600 nm.

The EL was estimated using the procedure provided by Sullivan & Ross [38], after immersing leaf disks in deionized water, electrical conductivity (EC1) was observed. The EC2 was determined by immersing the tubes in a water bath for 30 min at 45 °C and 55 °C, respectively. To determine the EC3, samples were further boiled at 100 °C for 10 min.

The EL values were determined using the following formulae:

(4) $$\mathrm{EL}\left(\%\right)=\frac{\left(\mathrm{EC}2-\mathrm{EC}1\right)}{\left(\mathrm{EC}3\right)}\times 100$$

### 2.9. Determination of Phenol, Flavonoids

In order to assess the phenolic content, 500 mg of dry leaf was homogenized in 80% ethanol. When the supernatant was centrifuged at 10,000 rpm for 10 min, it was treated with the reagent 100 μL Folin Ciocalteu (0.1 mL) immediately afterward. The sample’s absorbance was observed at 765 nm [39]. The method of Zhishen et al. [40] was used to assess the content of flavonoids. In this procedure, 500 μL of leaf extracts were combined with 300 μL of 5% NaNO_2_ and 450 μL deionized water to make a solution. In a subsequent 6 min period, 100 μL of 10% AlCl_3_ 6H_2_O was added. After 5 min, deionized water and 200 μL of 1 M NaOH were added to get the correct 10 mL mixture. After 5 min of incubation, the absorbance at 500 nm was measured with a spectrophotometer.

### 2.10. Determination of the Activity Levels of Antioxidant Enzymes

The leaf samples were ground in a pestle and mortar that had been pre-chilled to soak in phosphate buffer (100 mM, pH 7.8) containing thylene diaminetetra acetic acid (EDTA) (1 mM), polyvinyl pyrrolidine (1%), and phenylmethylsulfonyl fluoride (0.1 m). The samples were centrifuged at 12,000 rpm for 10 min at 4 °C. This was done to collect the supernatant for use in an enzyme assay. Superoxide dismutase (SOD) activity was determined by the ability of an enzyme extract to prevent the photochemical reduction of nitroblue tetrazolium [41]. The optical density at 560 nm was determined after incubating the assay mixture for 15 min under fluorescent illumination. In addition, the catalase (CAT) activity was determined by monitoring a reduction in optical density at 240 nm for 2 min while using H_2_O_2_ as a substrate, and the extinction coefficient (ε) of 0.036 mM^−1^ cm^−1^ was utilized for computation [42]. Furthermore, the method published by Nakano and Asada [43] was utilized to evaluate ascorbate peroxidase (APX) activity, and H_2_O_2_-dependent oxidation of ascorbate was detected as a change in absorbance at 290 nm for 2 min. Using the method described by Hori et al. [44], peroxidase (POD) activity was evaluated in a reaction mixture including enzyme extract phosphate buffer (50 mM, pH 7.0), 1 mM H_2_O_2_, and 1 mM guaiacol. For 3 min, the absorbance of the sample was measured at 470 nm. The activity was measured in enzyme unit mg^−1^ protein.

### 2.11. Statistical Analysis

Using the Statistix 8.1 software, the data were subjected to two-factor analysis of variance (ANOVA). The least significant differences (LSD) were used to compare means at the 0.05 probability level. Sigma plot software was used for data processing and plotting figures. Canoco 5 and GraphPad Prism 7 software were used to test the principal component analysis (PCA) and heat map analysis to differentiate between the different cultivars with different treatments.

## 3. Results

### 3.1. Seed Germination Behavior under Different Drought and Salinity Conditions

**Figure 1:**
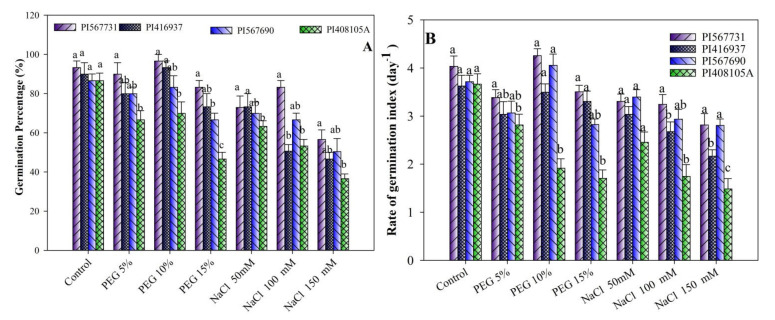
(**A**) Seed germination percentages (SGP) and (**B**) rate of germination indexes (GRI) of four soybean cultivars grown under drought and salinity stress conditions. Data presented are means ± SD. Different letters denote a significant difference at *p* < 0.05 based on the least significant difference (LSD) test. Letter a is highly significant than b, and ab means no significant differences between a with ab and b with ab.

**Figure 2:**
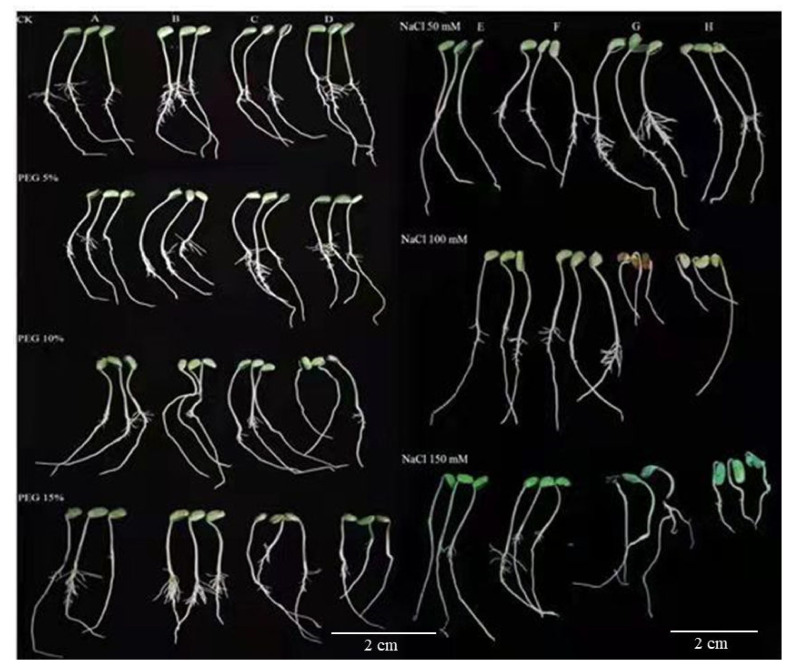
Seed germination of four soybean cultivars grown under different drought and salinity stress conditions. (**A**) PI31, (**B**) PI90, (**C**) PI37, (**D**) PI5A, (**E**) PI31, (**F**) PI90, (**G**) PI5A, (**H**) PI37.

Soybean seeds germination percentage (SGP) and germination rate index (GRI) were ominously impacted by drought and salinity stress in all cultivars (Figure 1A,B and Figure 2). A 10% PEG treatment resulted in a significant increase in the rate of seed germination compared to CK (Figure 1A,B and Figure 2). Soybean GP characteristics were modified by PEG content and cultivar; however, the interaction was not significant (Figure 1A,B). The GP was highest under the 10% drought treatment, followed by the 5% drought treatment, for all soybean cultivars compared to CK (Figure 1A,B and Figure 2, Table S2). Among the four genotypes, PI31, PI37, and PI90 resulted in better germination performances than the PI5A genotype. The PI31, PI37, and PI90 cultivars exhibited especially good performances. However, the GP decreased steadily with the increase in salt concentration except for PI31 (Figure 1A,B). In a control, the highest GP was recorded, but among the treatment groups, the highest GP was recorded with 100 mM of salt, followed by 50 mM and 150 mM (Figure 1A,B, Table S3).

Furthermore, the results revealed that the maximum GRI were at 5% and 10% of PEG (Figure 1A,B). However, at the 15% PEG level, the lowest GRI were found. The PI31 variety had the highest GRI among all varieties, followed by PI90 and PI37 varieties (PI5A having the lowest of all genotypes). As the PEG content increased, the GRI of all genotypes decreased (Figure 1A,B).

In addition, as the salt content increased, a gradual decrease in GRI was observed (*p* < 0.05). The GRI index was negatively affected by salinity levels (*p* < 0.05, Figure 1A,B); i.e., seed GRI steadily dropped in all varieties as salinity increased (Figure 1A,B, Table S3). The germination rate indexes of PI31 and PI90 were greater than those of PI5A and PI37 under normal conditions. In contrast, the GRI of PI31, PI90, and PI37 under salinity stress were considerably higher than that of PI5A (Figure 1A,B). The above results indicate substantial differences in the sensitivity of PI31, PI90, and PI5A to salinity, and that PI31 was more tolerant to salinity than PI37 (Figure 1A,B).

### 3.2. Germination Energy

**Figure 3:**
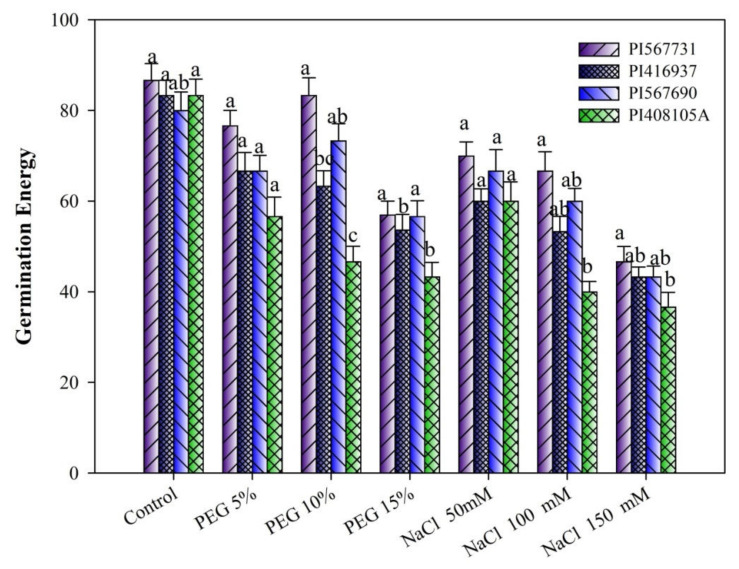
Germination energy of four soybean cultivars grown under drought and salinity stress conditions. Data presented are means ± SD. Different letters denote significant a difference at *p* < 0.05 based on the least significant difference (LSD) test. Letter a is highly significant than b, and ab means no significant differences between a with ab and b with ab.

PEG and NaCl treatments significantly impacted all cultivars’ GE, with PI31 having higher GE than the others in the PEG/NaCl treatment (Figure 3). The present results show that drought and salinity stress increments decreased GE in all cultivars. When all cultivars were exposed to PEG 15% and 150 mM NaCl, their GE was reduced dramatically, but there were no changes when exposed to PEG 10% and 50 mM NaCl. PI5A showed more germination energy retardation than PI31 under drought stress. However, under salinity stress, the lowest GE was observed in PI37. The results showed that the highest GE (Figure 3) was observed in PI31 at all PEG and NaCl levels. According to these findings, the degree of reduction was not the same for all soybean genotypes evaluated at the PEG and NaCl concentrations studied. A negative effect difference across all genotypes investigated for all germination traits, as shown in (Figure 3), indicates potential genetic variation in response to PEG-induced drought stress and salinity (Tables S2 and S3).

### 3.3. Plant Height and Biomass and RWC under Drought and Salinity Conditions

The results show that drought stress, salinity, and combined stress treatments reduced the plant height, biomass, and RWC (Table 2 and Tables S4–S7). The plant height of all cultivars was considerably reduced by drought stress and salinity. Under salinity treatment, the reduction was more in PI37 (64%) and less in PI31 (52%) than in control treatments. Under drought treatment, the reduction was greatest in PI5A (179.44%) and least in PI31 (49%), whereas it was intermediate in the other two varieties compared to the control treatments (Table 2).

The results show that combined stress further decreased the plant height by 126%, 140, 240%, and 252%, in PI31, PI37, PI90, and PI5A, respectively. Moreover, under salinity treatment, the lowest shoot fresh weight (SFW), shoot dry weight (SDW), and root dry weight (RDW) were recorded in PI37 (103%, 79%, and 60% of the control) and the highest in PI31 (101%, 87%, and 79% of the control). However, under drought treatment, the lowest SFW, SDW, and RDW were recorded in PI5A (67%, 68% and 69% of the control) and the highest in PI31 (61%, 13.99%, and 30.68% of the control). On average, 76%, 24%, 158%; and 76%, 130%, 10% of SFW, SDW, and RDW were recorded in the PI37 and PI90 soybean cultivars (Table 2). Similarly, combined stress treatments decreased SFW, SDW, and RDW (Table 2). The lowest SFW, SDW, and RDW were found in PI5A and the highest in PI31 under the combined treatment (Table 2 and Tables S4–S7).

### 3.4. Chlorophyll, Carotenoid, and Anthocyanin Content, and Chlorophyll Fluorescence

The results show that the Chl, carotenoids, anthocyanin contents, and the Chl fluorescence were reduced in all varieties due to drought stress, salinity, and combined stress treatments compared with the controls (Table 3 and Tables S4–S7). The results revealed that Chl a, Chl b, Chl (a + b), and carotenoid contents were significantly affected by drought, salinity, and combined treatments. Soybean cultivar PI31 had higher Chl a, Chl b, Chl (a + b), and carotenoid contents under drought, salinity, and combined stress treatments. In the others, compared to the control, Chl a, Chl b, Chl (a + b), and carotenoids were decreased due to combined stress treatment. The reductions were lower in PI31 (68%, 66%, 69%, and 70%) and higher in PI5A (89%, 70%, 61%, and 80%) compared to the control treatments. Furthermore, drought, salinity, and combined stress significantly reduced the anthocyanin content in all cultivars (Table 3). Under combined stress treatment, the respective contents were reduced by 39%, 51%, 46%, and 61% (Table 3 and Tables S4–S7).

**Figure 4:**
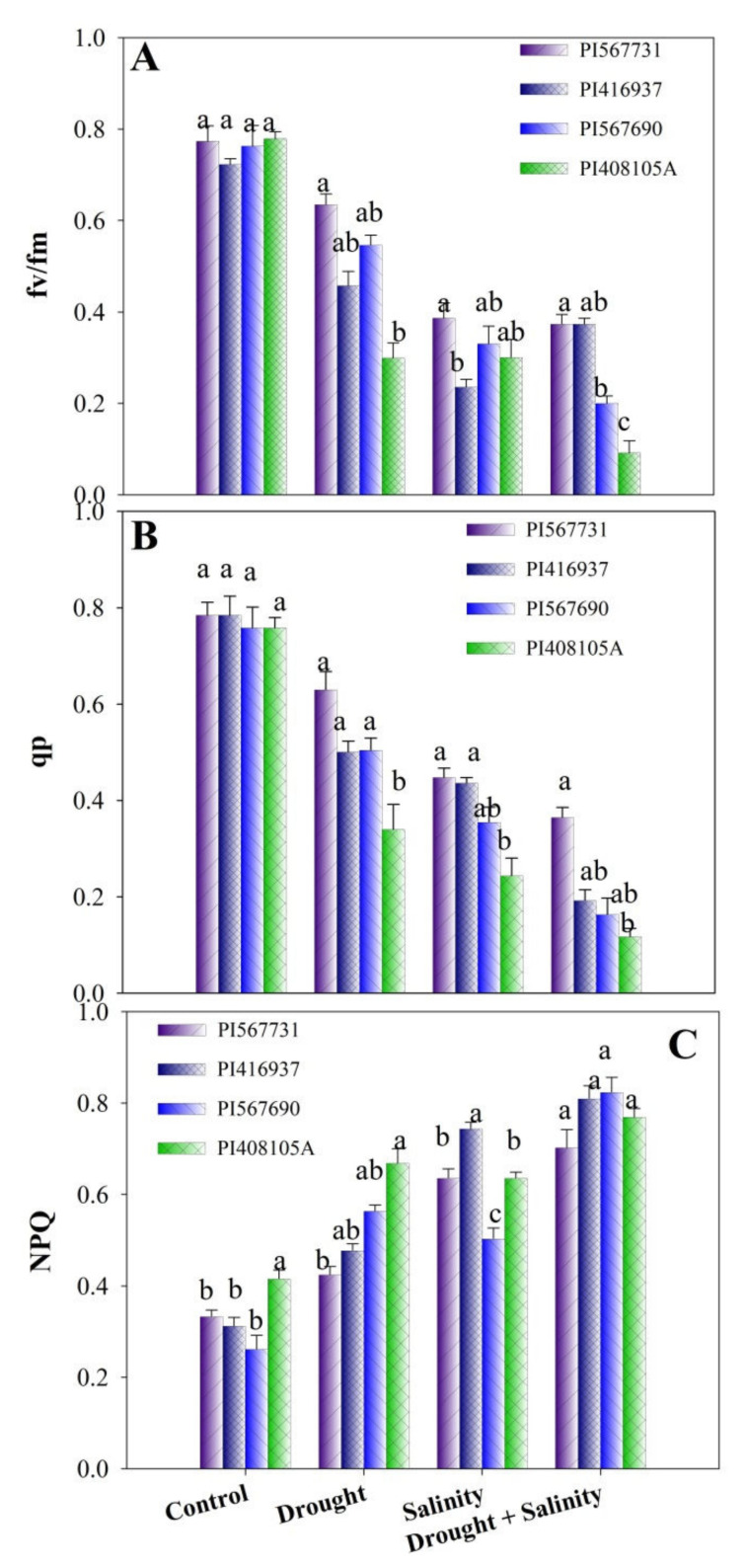
(**A**) Fv/Fm, (**B**) non-photochemical quenching (NPQ), and (**C**) photochemical quenching (qP) of four soybean cultivars grown under drought and salinity stress conditions. Data presented are means ± SD, and different letters denote a significant difference at *p* < 0.05 based on the least significant difference (LSD) test. Letter a is highly significant than b, and ab means no significant differences between a with ab and b with ab.

Moreover, the maximum yield of photosystem II (PSII, Fv/Fm) and photochemical quenching coefficient (qP) of soybean leaves reduced dramatically under drought, salinity, and combined stress, whereas the non-photochemical quenching coefficient (NPQ) increased (Figure 4A–C, Tables S4–S7). The Fv/Fm values evaluated under drought and salinity conditions were considerably reducedby 18% and 50%; 37% and 47%; 28% and 44%; and 62% and 57% in PI31, PI90, PI37, and PI5A, respectively, when compared with control treatment. The qP did not differ significantly in the CK and drought treatments, but the salinity and combined treatments reduced the qP. However, when compared to the control treatment, the salinity and drought treatments increased the qP in PI31 (Figure 4A–C). In all soybean cultivars, the NPQ in the drought and salinity treatment was considerably enhanced as compared to the control treatment. Compared to CK treatment, the NPQ in the combined treatment further increased in PI31, PI90, PI5A, and PI37 (Figure 4A–C, Tables S4–S7).

### 3.5. Sugars, Proline, Free Amino Acids, and Protein

**Figure 5:**
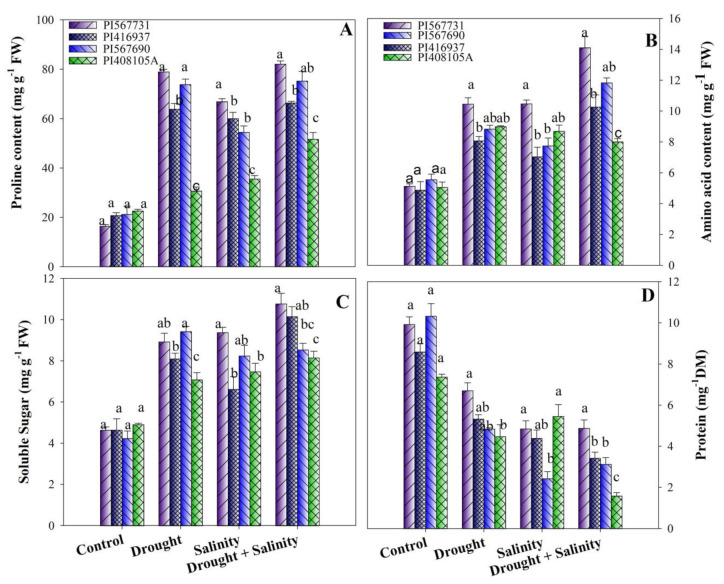
(**A**) Proline, (**B**) free amino acids, (**C**) soluble sugars, and (**D**) protein of four soybean cultivars grown under drought and salinity stress conditions. Data presented are means ± SD, and different letters denote a significant difference at *p* < 0.05 based on the least significant difference (LSD) test. Letter a is highly significant than b, and ab means no significant differences between a with ab and b with ab.

Under control conditions, all osmolytes (sugars, proline, and free amino acid) concentrations were statistically similar in all soybean varieties (Figure 5A–D, Tables S8–S11). However, drought, salinity, and combined stress significantly increased osmolytes’ accumulation in the soybean seedlings of all varieties. Soybean cultivar PI31 had by far the highest soluble sugar, proline, and free amino acid concentrations compared to the other cultivars under drought and salinity stress treatments, respectively (Figure 5A–D). Upon exposure to combined stress treatment, proline, sugar, and free amino acid contents increased by 399%, 125%, and 174% in PI31; 255%, 101%, and 113% in PI90; 128%, 73%, and 11% in PI05A; and 195%, 111%, and 110% in PI37, compared with the control conditions (Figure 5A–D). In addition, drought, salinity, and combined stress resulted in a substantial decrease in protein content in all soybean varieties compared to the control (Figure 5A–D, Tables S8–S11). Protein content decreased by 32%, 70%, 79%, and 60% in PI31, PI567690, PI5A and PI37, respectively, under combined stress conditions compared to the controls.

### 3.6. Total Phenolic and Flavonoid Contents

**Figure 6:**
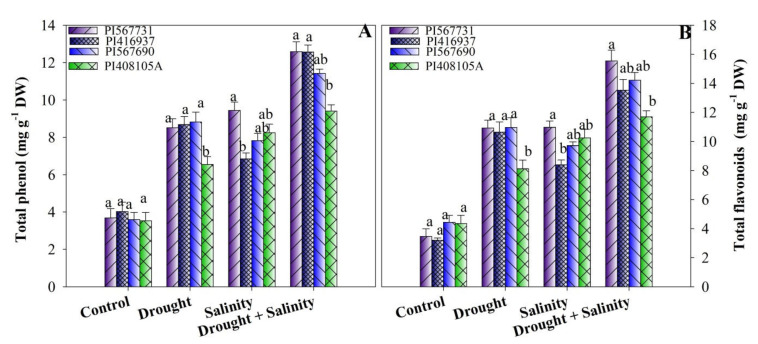
(**A**) Total phenols and (**B**) total flavonoids of four soybean cultivars grown under drought and salinity stress conditions. Data presented are means ± SD, and different letters denote a significant difference at *p* < 0.05 based on the least significant difference (LSD) test. Letter a is highly significant than b, and ab means no significant differences between a with ab and b with ab.

In this study, the accumulation of phenol and flavonoids was significantly augmented with drought, salinity, and combined stress treatments (Figure 6A,B, Tables S8–S11). Compared to the control, drought treated plants showed substantial improvements in their contents of phenolic compounds and flavonoids: 111% and 231.40% in PI31; 145% and 147% in PI90; 85% and 86% in PI5A; and 135% and 215% in PI37, respectively. Similar to drought treatment, the contents of total phenolic compounds and flavonoids were also increased under high salinity by 134% and 241%; 117% and 119%; 133% and 135%; and 86% and 152% in PI31, PI90, PI5A, and PI37 compared to the controls, respectively. Likewise, combined stress treatment further increased the phenol and flavonoid contents compared to the control (Figure 6A,B, Tables S8–S11).

### 3.7. Oxidative Stress Indicators

**Figure 7:**
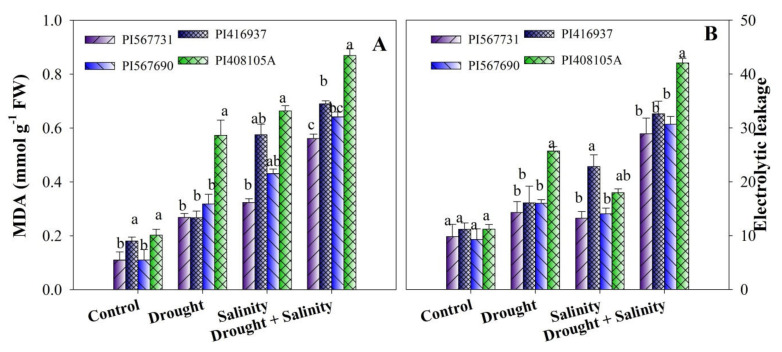
(**A**) Lipid peroxidation and (**B**) electrolyte leakage of four soybean cultivars grown under drought and salinity stress conditions. Data presented are means ± SD, and different letters denote a significant difference at *p* < 0.05 based on the least significant difference (LSD) test. Letter a is highly significant than b, and ab means no significant differences between a with ab and b with ab.

Under drought, salinity, and combined stress treatment, lipid peroxidation (MDA) and electrolyte leakage (EL) significantly (*p* ≤ 0.05) increased compared with the controls (Figure 7A,B, Tables S8–S11). Compared with its control, PI37 possessed higher MDA and EL contents under high salinity. Under drought stress treatment, PI5A has displayed the highest MDA and EL values. PI31 showed the least lipid peroxidation and electrolyte leakage under all stress conditions. Meanwhile, among the cultivars, PI37, PI90, and PI5A displayed maximum MDA and EL concentration reductions of 173%, 250%, and 174% and 281%, 686%, and 217%, respectively, compared to PI31 under combined stress conditions (Figure 7A,B).

### 3.8. Activity Levels of Antioxidant Enzymes

**Figure 8:**
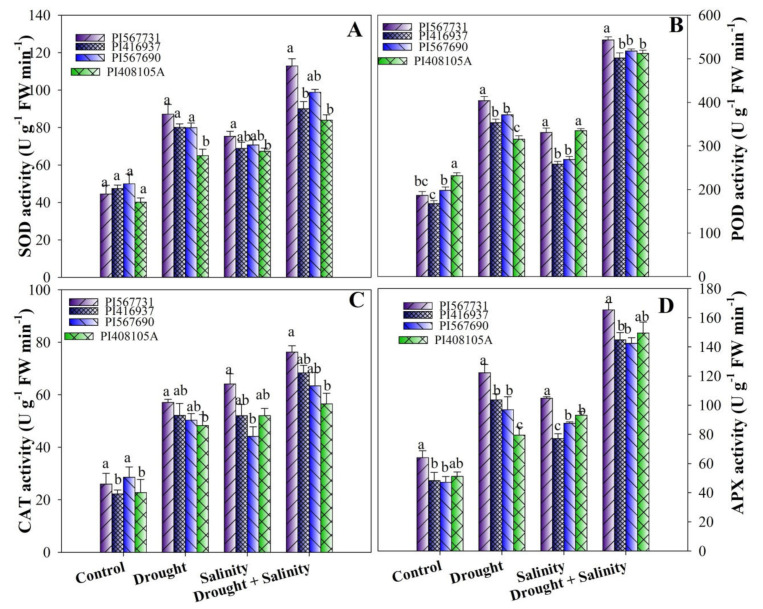
(**A**) Superoxide dismutase, (**B**) peroxidase, (**C**) catalase, and (**D**) ascorbate peroxidase activity levels of four soybean cultivars grown under drought and salinity stress conditions. Data presented are means ± SD, and different letters denote a significant difference at *p* < 0.05 based on the least significant difference (LSD) test. Letter a is highly significant than b, and ab means no significant differences between a with ab and b with ab.

Antioxidant enzymes’ activity levels, such as those of SOD, POD, CAT, and APX, were examined in soybean cultivars and found to significantly differ (Figure 8A–D, Tables S8–S11). Under separate drought and salinity treatments, SOD and CAT activity levels were significantly increased in PI31, PI37, PI90, and PI5A (Figure 8A–D). Plants exposed to combined stress treatment further augmented their activity, having highest improvements of 153%, 179%, 190%, and 90% in SOD; and 161%, 108%, 162%, 121% in CAT in PI31, PI90, PI37, and PI5A, respectively, compared to the controls. Moreover, in drought and salinity treatments, POD and APX activity levels increased in PI31, PI90, PI5A, and PI37 compared to their control treatments (Figure 8A–D). Likewise, combined stress further significantly increased the APX and POD activity levels by 83% and 206% in PI567731; 71% and 198% in PI90; 57% and 97% in PI5A; and 38% and 121% in PI37, respectively, compared to their controls (Figure 8A–D, Tables S8–S11).

### 3.9. Relationships among Physiological Traits under Drought Stress, Salinity, and Combined Stress Conditions

**Figure 9:**
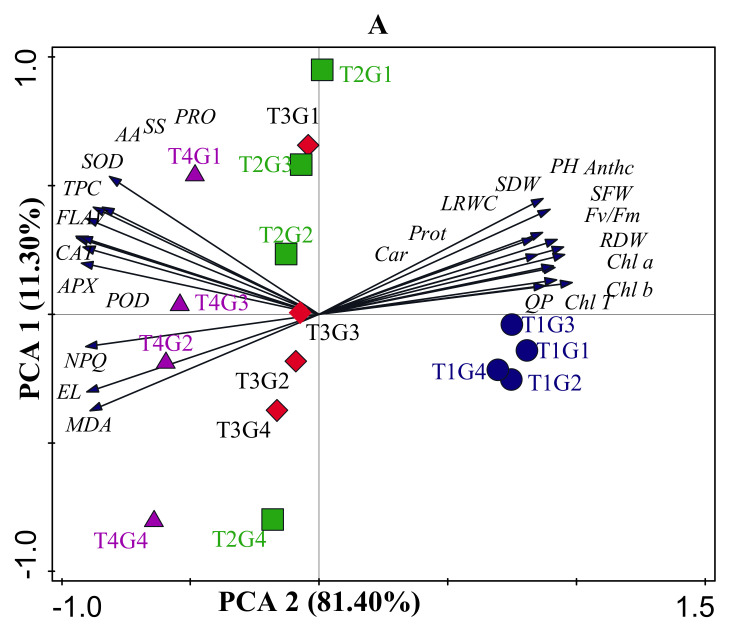

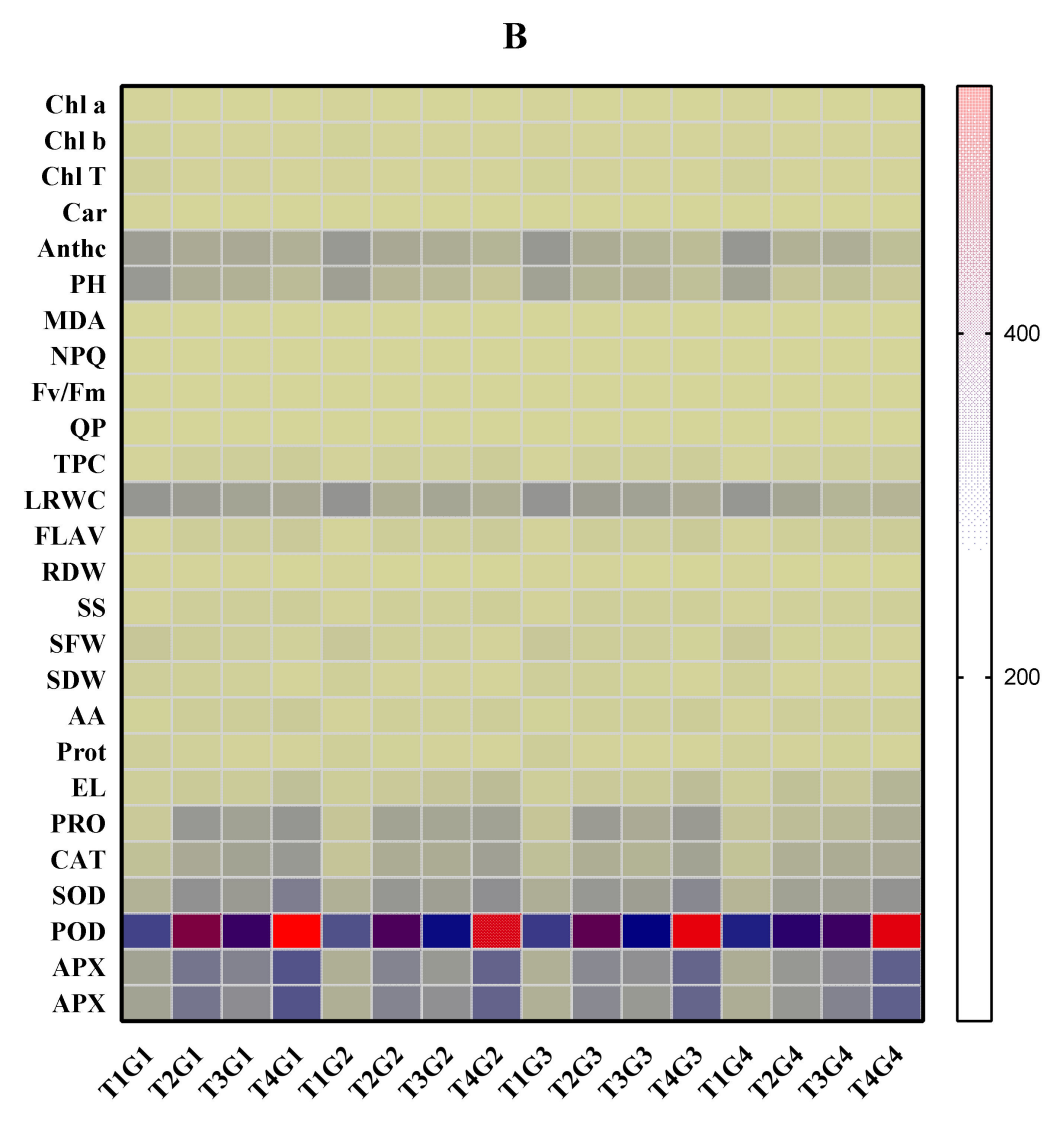
Multivariate statistical analysis indicates associations between treatments, variables, and cultivars. (**A**) Principal component analysis (PCA) based on eco-physiological traits in four soybean cultivars affected by drought, salinity, and combined stress. Blue circles, PI567731; green symbols, PI416937; red symbols, PI567690; and purple symbols, PI408105A in control, drought, salinity, and drought + salinity treatments; (**B**) Heatmap of correlation. T1, control: T2, drought: T3 salinity: T4 drought + salinity; G1, PI567731: G2, PI416937: G3, PI567690: G4, PI408105A. Chl: chlorophyll; Car: carotenoids Chl T: total chlorophyll; PH; plant height; SFW: shoot fresh weight: SDW: shoot dry weight: QP: photochemical quenching; NPQ: non-photochemical quenching; maximal photochemical efficiency (Fv/Fm), RLWC: relative leaf water content; MDA: malondialdehyde; EL: electrolyte leakage; PRO: proline; SS: soluble sugar; CAT: catalase; POD: peroxidase; SOD: superoxide dismutase; APX: ascorbate peroxidase; FLAV: flavonoid content; TPC: total phenolic content; AA: amino acid.

Across drought, salinity, and combined stress conditions, the principal component analysis (PCA) with two major components described 44% of the total variation in four different soybean cultivars PI31, PI90, PI5A, and PI37 (Figure 9A). The first PCA axis was used to differentiate no salinity treatments (well-watered and drought-stressed), salinity treatments (salinity, well-watered), and combined stress treatments (salinity + drought) (Figure 9A). Each treatment was segregated well under stressful conditions, and the four soybean cultivars were separated along the second PCA axis. Interestingly, the PCA results revealed that SOD, POD, carotenoid, APX, CAT, phenol, flavonoids, and total Chl were the significant contributors in PC1 and were strongly related to “PI31.” EL, MDA, and NPQ were substantially connected to “PI37” under salinity and “PI5A” under drought treatments (Figure 9A). Furthermore, “Chl a” was closely linked to “PI90.” The contents of Chl a and Chl b were found to be the most closely linked to “PI31” (Figure 9A).

### 3.10. Correlation Analysis in Soybean Cultivars under Drought Stress, Salinity, and Combined Stress Conditions

Furthermore, the heat map elucidates the physiological responses of the four soybean cultivars under various stress conditions, including drought, salinity, and combined stress (Figure 9B). According to our data, under drought, salinity and combined treatments, plant weights (SFW and SDW), Chl content, qp, RWC, protein, Fv/Fm activity, and proline content were correlated positively with secondary metabolites (phenol and flavonoids) and antioxidative enzymes (CAT, POD, APX, and SOD). The stress treatments are grouped apart from the other soybean treatments because of their increased NPQ, MDA, and EL contents.

## 4. Discussion

Environmental stresses, particularly drought and salinity stress, are the two most important abiotic factors limiting plants’ growth and productivity by restricting their physiological, biochemical, and molecular mechanisms, such as photosynthetic performance, metabolism of protein, and synthesis of lipids [7,45]. To date, most findings have focused on plant responses to each of these stressors separately [46], and these investigations have not provided predicted the strategies plants utilize to respond to combined stresses. Moreover, seedling growth may be a useful trait for the early detection of drought and salinity. Therefore, the present research explored the adverse effects of drought, salinity stress, and their combination on seed germination, growth, photosynthesis, and enzymatic antioxidation in soybeans.

Drought is well-known abiotic stress that causes major losses in plant growth, leading to considerable yield loss among many crops worldwide [6]. Many researchers have shown that simulating drought stress with PEG hypertonic solution is an effective tool during seed germination [47]. In this study, contrasting soybean cultivars were employed, including PI5A, PI31, PI90, and PI37 (Table 1), to evaluate the drought and salt resistance of the soybean germplasms and to comprehend the genetic influences on drought and salt stress tolerance. The present results showed that the seed germination attributes were enhanced by 10% PEG and decreased by 15% PEG treatments compared to the controls. A previous study reported that some plants could adapt to drought, and that low stress could enhance the seed germination, whereas high stress inhibited growth [48]. The effects of drought stress simulated by PEG on seed germination parameters, i.e., GP, GE, and GI, exhibited that seed germination attributes of soybean varied depending on the concentration of PEG600. The relative GP, GE, and GI were reduced as PEG concentrations increased [49]. We observed that 10% PEG could increase seed germination, but 15% PEG could greatly inhibit seed germination. Similar results to our findings were found in the study of Guo et al. [50]. Furthermore, salt concentration increases seed germination efficiency and decreases germination rate [51]. Salt stress can cause osmotic stress and ionic toxicity, which result in poor germination [52]. Our results showed that the seed germination performance was poor with a high salt concentration compared to the control conditions. Among the salinity treatments, he maximum germination performance was recorded at 100 mM NaCl, followed by 50 mM NaCl. The current findings are similar to those of Talei et al. [53].

Furthermore, a recent study stated that, during stressful situations, compared to seed germination, seedling growth is more affected [52]. The present findings demonstrate that salt and drought reduced plant fresh and dry weights in all soybean cultivars, but the responses differed. Due to drought stress, the decrease in plant biomass was greater in the sensitive cultivar (PI5A) than in the tolerant cultivar (PI31). In contrast, PI31 plants grown under combined stress exhibited produced more biomass than the others, indicating that PI567731 soybeans are the most drought and salinity tolerant of these cultivars. These findings demonstrate that the four genotypes have different growth strategies under stressful situations, which is consistent with recent findings [54,55]. In addition, according to previous research, plants can modify their growth distributions among roots and shoots to deal with stress conditions, such as water stress and salinity [56,57]. The present findings support previous research, as under drought, salinity, and combined stress, PI31 had more root biomass than the others (Table 2), which could be due to the roots’ enhanced nutrition and water adsorption capability. On the other hand, drought, salinity, and combined stress treatment reduced root biomass in PI37, PI90, and PI5A. This complex mode of growth trait retardation in different soybean genotypes under combined stress could be owing to distinct, particularly opposed signaling pathways caused through combined stress. [58].

Alexieva et al. [59] observed that a reduction in RWC was the primary cause of osmotic stress, which can also be caused by salt and drought. The sensitive PI5A cultivar was more influenced by the reduction in RWC than the PI31 and PI37 cultivars in our study under combined stress (Table 2). We found that when those cultivars were subjected to combined stress, they displayed different levels of sensitivity. Additionally, to determine the performance of their photosynthetic systems under drought and salinity stress conditions, the photosynthetic pigments and Chl fluorescence were measured after drought, salinity, and combined stress treatments. Results showed that drought and salinity stress considerably affected the photosynthetic parameters in the soybean leaves, but the reductions were not similar in the cultivars (Table 3; Figure 4A–C). Generally, alterations in Fv/Fm are frequently corroborated by changes in the concentrations of particular photosynthetic pigments and cell structure modulations, which can be affected by a wide range of environmental factors, including water deficit, nutritional status, and temperature, that affect PSII activity [60]. In the present study, we found that drought and combined stress more adverse effects on the Fv/Fm of the drought-sensitive cultivar (PI5A) than the Fv/Fm of the drought-tolerant cultivars (PI31 and PI37) (Figure 4A–C), demonstrating that PI408105A is more sensitive to drought stress than the other cultivars. On the other hand, salinity stress has a more severe adverse effect on the Fv/Fm of the salt-sensitive cultivar (PI37) than the Fv/Fm of the other cultivars. A recent study has shown that drought can alter the reaction center and impede the electron transport system, decreasing the Fv/Fm [61]. Photosynthetic pigment concentrations, such as those of Chl a, Chl b, and Car, are widely employed to directly check the photosynthesis capabilities of plants in stressful situations [62]. Reductions in Chl and Car concentration have previously been documented to cause significant photosynthetic capacity impairments [63]. In this study, Chl content was observed to be reduced in all stress treatments, and the reductions in Chl in salt treatment groups were more severe than in drought treatment groups (Table 3). In addition, significant reductions in all Chl pigments occurred after seven days of salt stress treatment, demonstrating that salt stress disrupted the synthesis and expedited the breakdown of leaf pigments. Previous studies on hybrid *Pennisetum* [64], *Populus euphratica*, and *P. pruinosa* seedlings [57], supported this hypothesis when salt-treated plants were subjected to drought stress. Their Chl a, Chl b, total Chl, and carotenoid contents were reduced significantly compared to the control plants.

Plants also accumulate a wide range of organic solutes in response to external osmotic impending changes to deal with environmental factors. Proline, an organic solute, is well-known for its osmotic adaptation activity and role in stress responses enhancement by inhibiting cellular membranes and enzyme integrity [65]. According to our results, stress-treated plants had significantly higher proline content than non-treated plants. Furthermore, plants treated with 15% PEG and 200 mM stress exhibited similar adjustments to salt stress when proline levels were nearly equal. Previous findings demonstrated that plant survival, stress tolerance, and biochemical changes under stressed conditions, such as drought and salinity, are all dependent on non-structural carbohydrates [66,67]. Several studies have found that sugars play a vital role in plants’ osmotic regulation, including cell turgor maintenance, and absorption and transportation of water under stress [67,68]. In the present study, under combined stress treatment, PI31, PI90, and PI37 soybean plants had higher leaf soluble sugar contents than their controls (Figure 5A–D), indicating that PI31, PI90, and PI37 soybeans may have superior osmotic regulation. Besides this, free amino acids (FAA) are involved in osmotic regulation, helping cells maintain their osmotic potential [69]. We observed that, under all stressful conditions, PI31 and PI90 accumulated more FAA than other soybeans used in this study. Therefore, PI31 and PI90 are more efficient in osmotic regulation, maintaining cell osmotic potential, and water absorption under stressful conditions than the other cultivars (Figure 5A–D).

Furthermore, phenols and flavonoids are crucial for improving cellular homeostasis during drought and salt stress. The amounts of phenolic compounds and flavonoids in our study increased as the concentrations of NaCl and PEG increased. Increased phenolic and flavonoid contents in drought and salt-treated plants could increase ROS production. Increased phenolic and flavonoid contents could also be helpful due to their scavenging activity, protecting plants from ROS by deactivating the free radicals, quenching the ROS, and the decomposing peroxides that are ultimately generated during stress. Some medicinal and aromatic plants, such as rosemary, basil, and pennyroyal [70], have shown that phenolic content increases as stress concentration increases. Surprisingly, in *Nigella sativa* [71], phenolic content decreases as the salt concentration rises.

Environmental conditions such as drought and high salinity lead to ROS production, leading to lipid peroxidation, antioxidant deprivation, and eventually, gene expression alterations [72]. The present investigation found that all soybean cultivars had greater ROS concentrations during drought and salinity stress and combined stress; however, the EL content was highest in the sensitive cultivars (PI5A) under drought and combined treatments and PI37 under salinity and combined treatments, implying higher oxidative stress under stressed conditions. The generation of ROS has been proposed as a major symptom of phytotoxicity, and this process has been extensively studied in plants in various environmental conditions [73,74]. Malondialdehyde (MDA), a key lipid peroxidation product, is linked to oxidative damage in severe stress situations [75]. Our data demonstrated that drought and salinity augmented lipid peroxidation levels in all soybean cultivars, indicating greater oxidative injuries [76], although PI37 and PI5A had more severe oxidative damage than other soybeans. These results revealed that PI31 had less damage to MDA than the other cultivars when exposed to drought, salinity, and combined stress treatments. Antioxidant enzymes are essential for scavenging superoxide ions and lipid peroxidation tolerance. According to previous research, stressful environments can cause substantial increases in the activity of enzymes such as POD, SOD, CAT, and APX, which are well associated with the scavenging ability of ROS and serve as essential protective measures to deal with stressful environmental factors [12,77]. SOD is the first enzyme in the antioxidant system, and it transforms the highly reactive OH^•^ radical and superoxide (O_2_^•−^) to less harmful H_2_O_2_, reducing DNA, protein, and damage to the membrane [78]. The enzyme activity levels in all soybeans increased dramatically in this study. Under drought stress, the SOD, POD, CAT, and APX activity levels were higher in PI31, PI90, and PI37 than in PI5A. At the same time, POD contents were lower in PI37 and greater in PI5A in terms under high salinity. Under combined stress conditions, all four showed higher levels of SOD and POD APX, and CAT as compared to their controls (Figure 8A–D). In summary, PI31 and PI90 appear to have highly effective antioxidant defense mechanisms to cope with stress, explaining their higher stress tolerance. These results are in agreement with other findings: other authors have also found enhanced antioxidant activity in alfalfa [79], maize [80], sugarcane species [15], *Populus yunnanensis* [81], *T. aestivum* [82], *Populus euphratica*, and *P. pruinosa* [57] under stress. Overall, the antioxidant enzyme complex’s improved efficacy found under stress conditions could be linked to tolerance mechanisms based on fine-tuned redox state management.

## 5. Conclusions

The responses of different soybean cultivars to drought and salinity stress were evaluated at the germination and seedling stages. Under drought treatment, compared to the control and 15% PEG treatment, seed germination was improved with the 10% PEG treatment. However, seed germination was reduced under salt treatment as the salt concentration increased. Furthermore, the present investigation revealed that under drought, salinity, and combined stress especially, PI37 and PI5A soybeans suffered more severe inhibitory effects and showed less tolerance than PI31 soybeans. Under stressful conditions, the antioxidant enzyme activity and osmoregulation of the PI31 soybean were higher, and the ROS accumulation was lower than in PI37 and PI5A. The present information collectively demonstrates that the PI31 soybean is more stress-tolerant whether exposed to drought stress, salt, or both. The results of this study are novel and advance our understanding of the plant responses to drought, salt, and combined stressors. These findings are especially valuable for cultivation management due to the frequency of droughts and worsened soil salinity in many areas. Having a better understanding of the specific responses and tolerances to drought and salinity of soybean cultivars is critical and will support environmental movements to re-green agricultural lands.

## Figures and Tables

**Table 1:** Details of soybean cultivars used in the study.

Sl. No.	Accession Name of the Cultivar	Cultivar	Origin/Country	Stress Tolerance	References
1	PI5A	KAS 633-19	Korea, South	Flooding tolerance	[27]
2	PI31	Fu yang (56)	China	Drought tolerance	[26]
3	PI90	Fu yang (7)	China	Drought tolerance	[25]
4	PI37	Houjaku Kuwazu	Japan	Aluminum-resistant	[23]

**Table 2:** Plant height, shoot fresh weight, shoot dry weight, root dry weight, and leaf relative water content (LRWC) of four soybean cultivars grown under drought and salinity stress conditions. Data presented are means ± SD. Different letters denote a significant difference at *p* < 0.05 based on the least significant difference (LSD) test.

Cultivars	Treatments	Plant Height (cm)	Shoot Fresh Weight (mg g^−1^ FW)	Shoot Dry Weight (mg g^−1^ FW)	Root Dry Weight (mg g^−1^ FW)	LRWC (%)
PI31	Control	76.663 ± 0.95 a	19.603 ± 0.65 a	8.937 ± 0.30 a	3.297 ± 0.40 a	81.376 ± 2.37 a
	Drought	51.410 ± 3.20 a	12.173 ± 0.37 a	7.840 ± 0.32 a	2.217 ± 0.06 a	73.443 ± 2.55 a
	Salinity	45.840 ± 0.76 a	9.743 ± 0.36 a	7.037 ± 0.76 a	1.55 ± 0.28 a	63.09 ± 2.62 a
	drought + Salinity	33.93 ± 1.06 a	6.800 ± 0.46 a	3.270 ± 0.03 a	1.043 ± 0.07 a	58.45 ± 1.56 a
PI90	Control	66.567 ± 2.27 b	18.233 ± 0.32 a	9.433 ± 0.65 a	3.20 ± 0.07 a	83.119 ± 2.57 a
	Drought	43.91± 1.02 b	10.300 ± 0.34 a	7.633 ± 0.55 ab	1.280 ± 0.55 b	70.96 ± 1.89 a
	Salinity	40.85 ± 2.40 ab	8.933 ± 0.40 a	5.27 ± 0.17 b	0.920 ± 0.18 b	65.831 ± 1.57 a
	Drought + Salinity	27.68 ± 0.76 b	5.657 ± 0.18 ab	2.867 ± 0.18 ab	0.727 ± 0.18 ab	55.514 ± 2.81 ab
PI37	Control	69.233 ± 1.12 ab	18.100 ± 0.22 a	8.833 ± 0.22 a	3.100 ± 0.11 a	83.92 ± 3.21 a
	Drought	39.43 ± 1.06 b	7.927 ± 0.30 b	4.143 ± 0.64 b	1.190 ± 0.14 bc	50.5 1± 1.27 b
	Salinity	36.267 ± 1.20 b	6.7733 ± 0.38 b	4.410 ± 0.46 b	1.243 ± 0.12 ab	60.79 ± 1.35 a
	Drought + Salinity	20.367 ± 0.03 c	4.680 ± 0.20 b	2.263 ± 0.26 b	0.497 ± 0.26 c	48.47 ± 2.09 bc
PI5A	Control	62.93 ± 2.90 b	16.74 ± 0.90 a	8.267 ± 0.40 a	2.897 ± 0.08 a	80.39 ± 4.44 a
	Drought	22.03 ± 1.11 c	5.480 ± 0.30 c	2.573 ± 0.33 c	0.887 ± 0.03 c	61.78 ± 3.52 ab
	Salinity	25.43 ± 1.93 c	6.28 ± 0.23 b	4.023 ± 0.86 c	1.030 ± 0.08 ab	42.48 ± 2.45 b
	Drought + Salinity	17.33 ± 1.40 c	4.657 ± 0.23 b	1.633 ± 0.22 c	0.347 ± 0.22 c	41.39 ± 1.53 c

**Table 3:** Leaf chlorophyll pigments, carotenoid contents, and anthocyanin contents of four soybean cultivars grown under drought and salinity stress conditions. Data presented are means ± SD, and different letters denote a significant difference at *p* < 0.05 based on the least significant difference (LSD) test.

Cultivars	Treatments	Chl a (mg g^−1^ FW)	Chl b(mg g^−1^ FW)	Total Chl (mg g^−1^ FW)	Carotenoid (mg g^−1^ FW)	Anthocyanin (μg g^−1^ FW)
PI31	Control	1.414 ± 0.67 a	5.278 ± 0.16 a	6.692 ± 1.09 a	1.283 ± 0.16 a	71.490 ± 1.9 a
	Drought	0.816 ± 0.015 a	3.202 ± a 0.18 a	4.018 ± 1.21 a	0.476 ± 0.05 a	55.385 ± 4.46 a
	Salinity	0.774 ± 0.02 a	2.964 ± 0.2 a	3.738 ± 0.72 a	0.475 ± 0.22 a	56.956 ± 1.87 a
	Drought + Salinity	0.449 ± 0.04 a	1.982 ± 0.19 a	2.431 ± 0.12 a	0.451 ± 0.34 a	48.267 ± 3.24 a
PI37	Control	1.388 ± 0.88 a	5.149 ± 0. 10 a	6.537 ± 0.43 a	0.944 ± 0.27a	76.596 ± 1.12 a
	Drought	0.625± 0.03 a	2.380 ± 0.09 bc	3.005 ± 0.64 b	0.2887 ± 0.18 ab	57.676 ± 1.33 a
	Salinity	0.475 ± 0.01 b	2.231± 0.36 b	2.706± 0.57c	0.256 ± 0.45 b	42.815 ± 4.48 b
	Drought + Salinity	0.231± 0.07 b	1.728 ± 0.17 ab	1.959 ± 0.54c	0.174 ± 0.33 bc	32.144 ± 4.67 b
PI90	Control	1.437 ± 0.44 a	4.994 ± 0.14 a	6.431 ± 1.13 a	1.0172 ± 1.11a	78.691 ± 2.85 a
	Drought	0.316 ± 0.06 b	2.771 ± 0.28 ab	3.087 ± 0.74 a	0.337 ± 0.55 ab	52.544 ± 0.79 ab
	Salinity	0.251 ± 0.08 c	2.883 ± 0.19 a	3.134 ± 0.22 b	0.423 ± 0.13 ab	51.784 ± 2.74 ab
	Drought + Salinity	0.114 ± 0.03 b	1.922 ± 0.11 a	2.063 ± 0.29 b	0.272 ± 0.65 b	42.750 ± 3.53 ab
PI5A	Control	1.388 ± 0.055a	4.902 ± 0.29 a	6.29 ± 1.08 b	0.967 ± 0.88 a	80.262 ± 6.17 a
	Drought	0.215± 0.03b	2.225 ± 0.15 c	2.44 ± 0.98b	0.181 ± 0.57 b	46.94 ± 3.88 b
	Salinity	0.233 ± 0.21c	2.356 ± 0.17 b	2.589 ± 0.65c	0.254 ± 0.14 b	50.082 ± 5.57 ab
	Drought + Salinity	0.158 ±0.14 b	1.421 ± 0.12 b	1.579 ± 0.54ab	0.1284 ± 0.44 c	30.311 ± 0.74 b

## Data Availability

Data is contained within the article.

## Supplementary Materials

The following supporting information can be downloaded at: https://www.mdpi.com/article/10.3390/antiox11030498/s1, Table S1: Different drought and salt treatments which used for imposing stress for 7days; Table S2. Seed germination percentage (SGP), rate of germination index (GRI) and Germination energy (GE) of four soybean cultivars grown under drought stress conditions. Data presented are means ± SD. Different letters denote significant difference at *p* < 0.05 based on the least significant difference (LSD) test. Abbreviation; CK = control, D1 = PEG6000 5% Drought; D2 = PEG6000 10% Drought; D3, PEG6000 15% Drought; Table S3. Seed germination percentage (SGP), rate of germination index (GRI) and Germination energy (GE) of four soybean cultivars grown under Salinity stress conditions. Data presented are means ± SD. Different letters denote significant difference at *p* < 0.05 based on the least significant difference (LSD) test. Abbreviation; CK = control, S1 = NaCL 50 mM; S2 = NaCL 100 mM; S3 = NaCL 150 Mm; Table S4. Plant height, Shoot fresh weight, Shoot dry weight, Root dry weight, and leaf relative water content (LRWC), Leaf chlorophyll pigments, carotenoid contents, and Anthocyanin contents, Fv/Fm, non-photochemical quenching (NPQ) and photochemical quenching (qP) of PI567731 soybean cultivars grown under drought and salinity stress conditions. Data presented are means ± SD. Different letters denote significant difference at *p* < 0.05 based on the least significant difference (LSD); Table S5. Plant height, Shoot fresh weight, Shoot dry weight, Root dry weight, and leaf relative water content (LRWC), Leaf chlorophyll pigments, carotenoid contents, and Anthocyanin contents, Fv/Fm, non-photochemical quenching (NPQ) and photochemical quenching (qP) of PI416937 soybean cultivars grown under drought and salinity stress conditions. Data presented are means ± SD. Different letters denote significant difference at *p* < 0.05 based on the least significant difference (LSD); Table S6. Plant height, Shoot fresh weight, Shoot dry weight, Root dry weight, and leaf relative water content (LRWC), Leaf chlorophyll pigments, carotenoid contents, and Anthocyanin contents, Fv/Fm, non-photochemical quenching (NPQ) and photochemical quenching (qP) of PI567690 soybean cultivars grown under drought and salinity stress conditions. Data presented are means ± SD. Different letters denote significant difference at *p* < 0.05 based on the least significant difference (LSD); Table S7. Plant height, Shoot fresh weight, Shoot dry weight, Root dry weight, and leaf relative water content (LRWC), Leaf chlorophyll pigments, carotenoid contents, and Anthocyanin contents, Fv/Fm, non-photochemical quenching (NPQ) and photochemical quenching (qP) of PI408105A soybean cultivars grown under drought and salinity stress conditions. Data presented are means ± SD. Different letters denote significant difference at *p* < 0.05 based on the least significant difference (LSD); Table S8. Proline, Free Amino acids, Soluble Sugars, Protein, Total phenols, Total flavonoids, lipid peroxidation and Electrolyte leakage, Superoxide dismutase, Peroxidase, Catalase, and Ascorbate peroxidase of PI567731 soybean cultivars grown under drought and salinity stress conditions. Data presented are means ± SD. Different letters denote significant difference at *p* < 0.05 based on the least significant difference (LSD); Table S9. Proline, Free Amino acids, Soluble Sugars, Protein, Total phenols, Total flavonoids, lipid peroxidation and Electrolyte leakage, Superoxide dismutase, Peroxidase, Catalase, and Ascorbate peroxidase of PI416937 soybean cultivars grown under drought and salinity stress conditions. Data presented are means ± SD. Different letters denote significant difference at *p* < 0.05 based on the least significant difference (LSD); Table S10. Proline, Free Amino acids, Soluble Sugars, Protein, Total phenols, Total flavonoids, lipid peroxidation and Electrolyte leakage, Superoxide dismutase, Peroxidase, Catalase, and Ascorbate peroxidase of PI567690 soybean cultivars grown under drought and salinity stress conditions. Data presented are means ± SD. Different letters denote significant difference at *p* < 0.05 based on the least significant difference (LSD); Table S11. Proline, Free Amino acids, Soluble Sugars, Protein, Total phenols, Total flavonoids, lipid peroxidation and Electrolyte leakage, Superoxide dismutase, Peroxidase, Catalase, and Ascorbate peroxidase of PI408105A soybean cultivars grown under drought and salinity stress conditions. Data presented are means ± SD. Different letters denote significant difference at *p* < 0.05 based on the least significant difference (LSD).
