# Supplementary material for: Seed Germination Behavior, Growth, Physiology and Antioxidant Metabolism of Four Contrasting Cultivars under Combined Drought and Salinity in Soybean
Source: Antioxidants (Basel). 2022 Mar 3;11(3):498. doi: 10.3390/antiox11030498 (PMC8944481; doi:10.3390/antiox11030498)
Supplement: Supplementary file 1 [file antioxidants-11-00498-s001.zip › antioxidants-1548622-supplementary.pdf]

Table S1. Different drought and salt treatments which used for imposing stress for 7days.

S.No	Treatments	PEG/Salt concentration
1	CK	Control condition
2	Drought	15% PEG-6000
3	Salinity	150 mM NaCl
4	Combined Stress	15% PEG-6000 + 150 mM NaCl

Table S2. Seed germination percentage (SGP), rate of germination index (GRI) and Germination energy (GE) of four soybean cultivars grown under drought stress conditions. Data presented are means ± SD. Different letters denote significant difference at $p < 0.05$ based on the least significant difference (LSD) test. Abbreviation; CK=control, D1=PEG6000 5% Drought; D2= PEG6000 10% Drought; D3, PEG6000 15% Drought.

Cultivars	Variables	Treatments					
		Control VS D 1		Control VS D2		Control VS D3	
		CK	D1	CK	D2	CK	D3
PI567731	SGP	93.33 ±3.33 a	90 ± 5.35 a	93.33 ±3.33 a	96.67 ± 3.33 a	93.33 ±3.33 a	83.33 ± 3.33 a
	GRI	4.04 ± 0.59 a	3.39 ± 0.16 b	4.04± 0.59 a	4.26 ± 0.34 a	4.04± 0.59 a	3.51 ± 0.13 a
	GE	86.67 ± 3.59a	76.67 ± 3.33 a	86.67± 3.59a	83.33 ± 6.87 a	86.67± 3.59 a	57.00 ± 3.00 b
PI416937	GP	90 ± 5.77 a	80.00 ± 5.75 a	90 ± 5.77 a	93.33 ±3.33 a	90 ± 5.77 a	73.33 ± 6.67 a
	GRI	3.63 ± 0.13 a	3.04 ± 0.29 a	3.63 ± 0.13 a	3.50 ± o.43 a	3.63 ± 0.13 a	3.31 ± 0.31 a
	GE	83.33 ± 5.33 a	66.67 ± 4.26 a	83.33 ± 5.33 a	63.33± 3.37 a	83.33 ± 5.33 a	53.67± 0.41 b
PI567690	GP	86.67 ± 3.33 a	80.00 ± 3.33 a	86.67 ±3.33 a	83.33 ±5.75 a	86.67 ±3.33 a	66.67 ±3.33 a
	GRI	3.72 ± 0.22 a	3.07 ± 0.26 a	3.72 ± 0.22 a	4.60 ± 0.37 a	3.72 ± 0.22 a	2.83 ± 0.41 a
	GE	80.00 ± 5.77 a	66.67 ± 4.42 a	80.00 ± 5.77 a	73.33 ± 5.77 a	80.00 ± 5.77 a	56.67 ± 3.43 a
PI408105A	GP	86.67 ± 3.77 a	66.67± 4.66 a	86.67 ±3.77	70 ± 5.77 b	86.67 ±3.77a	46.667 ± 3.33 a
	GRI	3.67 ± 0.31 a	2.82 ± 0.32a	3.67 ± 0.31 a	1.92 ± 0.59a	3.67 ± 0.31 a	1.71 ± 0.17 b
	GE	83.33 ± 6.67 a	56.67 ±8.22a	83.33 ± 6.67 a	46.67 ± 3.35 a	83.33 ± 6.67 a	43.33± 3.17 b

Table S3. Seed germination percentage (SGP), rate of germination index (GRI) and Germination energy (GE) of four soybean cultivars grown under Salinity stress conditions. Data presented are means ± SD. Different letters denote significant difference at $p < 0.05$ based on the least significant difference (LSD) test. Abbreviation; CK=control, S1=NaCL 50 mM; S2=NaCL 100 mM; S3=NaCL 150 mM.

**Cultivars**	**Variables**	**Treatments**					
		Control VS S 1		Control VS S2		Control VS S3	
		CK	S1	CK	S2	CK	S3
PI567731	GP	93.33 ± 5.66 a	73 ± 5.77 a	93.33 ± 5.66 a	83.33 ±3.33 a	93.33 ± 5.66 a	56.67 ± 4.81 b
	GRI	3.8333 ± 0.21 a	3.31 ± 0.25 a	3.83 ± 0.21 a	3.25 ± 0.22 a	3.8333 ± 0.21 a	2.82 ± 0.33 a
	GE	80.00 ± 2.77 a	70 ± 5.77 a	80.00 ± 2.77 a	66.67 ± 6.22 a	80.00 ± 2.77 a	46.67 ± 3.33 b
PI416937	GP	96.67 ± 3.33 a	73.33 ± 6.67 a	96.67 ± 3.33 a	50.67 ± 3.33 b	96.67 ± 3.33 a	46.67 ± 3.33 b
	GRI	3.63 ± 0.29 a	3.40 ± 0.45 a	3.63 ± 0.29 a	2.92 ± 0.2.81 a	3.63 ± 0.29 a	2.81 ± 0.33 a
	GE	80.00 ± 3.33 a	60.00 ± 2.72 a	80.00 ± 3.33 a	53.33 ± 3.33 a	80.00 ± 3.33 a	43.33 ± 2.13 b
PI567690	GP	90 ± 5.67 a	70 ± 5.77 a	90 ± 5.67 a	66.67 ± 3.33 a	90 ± 5.67 a	50.44 ± 6.66 b
	GRI	3.59 ± 0.33 a	3.04 ± 0.60 a	3.59 ± 3.33 a	2.68 ± 0.20 b	3.59 ± 3.33 a	2.17 ± 0.13 b
	GE	83.33 ±5.77a	66.67 ± 6.67a	83.33 ± 5.77a	60.00 ± 2.81 a	83.33 ± 5.77a	43.33 ± 2.33 b
PI408105A	GP	90 ± 3.33 a	63.33 ± 2.82 a	90 ± 3.33 a	53.33 ± 3.33 a	90 ± 3.33 a	36.63 ± 2.33 b
	GRI	3.56 ± 0.34 a	2.46 ± 0.25 b	3.56 ± 0.34 a	1.75 ± 0.28 b	3.56 ± 0.34 a	1.49 ± 0.21 b
	GE	90 ± 5.77 a	60.00 ± 5.25 a	90 ± 5.77 a	40.00 ± 2.28 b	90 ± 5.77 a	36.67 ± 0.21 b

Table S4. Plant height, Shoot fresh weight, Shoot dry weight, Root dry weight, and leaf relative water content (LRWC), Leaf chlorophyll pigments, carotenoid contents, and Anthocyanin contents, Fv/Fm, non-photochemical quenching (NPQ) and photochemical quenching (qP) of PI567731 soybean cultivars grown under drought and salinity stress conditions. Data presented are means ± SD. Different letters denote significant difference at p < 0.05 based on the least significant difference (LSD).

**Cultivar**	**PI567731**					
	Treatments					
	Control VS Drought		Control VS Salinity		Control VS drought salinity	
Variables	Control	Drought	Control	salinity	Control	drought + salinity
plant height (cm plant-1)	76.663 ± 0.95 a	51.410 ± 3.20 b	76.663 ± 0.95 a	45.840 ± 0.76 b	76.663 ± 0.95 a	33.93 ± 1.06 b
Shoot fresh weight (mg g-1 FW)	19.603 ± 0.65 a	12.173 ± 0.37 b	19.603 ± 0.65 a	9.743 ± 0.36 b	19.603 ± 0.65 a	6.800 ± 0.46 b
Shoot dry weight (mg g-1 FW)	8.937 ± 0.30 a	7.840 ± 0.32 b	8.937 ± 0.30 a	7.037 ± 0.76 b	8.937 ± 0.30 a	3.270 ± 0.03 b
Root dry weight (mg g-1 FW)	3.297 ± 0.40 a	2.217 ± 0.06 b	3.297 ± 0.40 a	1.55 ± 0.28 b	3.297 ± 0.40 a	1.043 ± 0.07 b
LRWC (%)	81.376 ± 2.37 a	73.443 ± 2.55 b`	81.376 ± 2.37 a	63.09 ± 2.62 b	81.376 ± 2.37 a	58.45 ± 1.56 b
Chl a (mg g^{-1} FW)	1.414 ± 0.67 a	0.816 ± 0.015 b	1.414 ± 0.67 a	0.774 ± 0.02 b	1.414 ± 0.67 a	0.449 ± 0.04 b
Chl b (mg g^{-1} FW)	5.278 ± 0.16 a	3.202 ± 0.18 b	5.278 ± 0.16 a	2.964 ± 0.2 b	5.278 ± 0.16 a	1.982 ± 0.19 b
Total Chl (mg g^{-1} FW)	6.692 ± 1.09 a	4.018 ± 1.21 b	6.692 ± 1.09 a	3.738 ± 0.72 b	6.692 ± 1.09 a	2.431 ± 0.12 b
Carotenoid (mg g^{-1} FW)	1.283 ± 0.16 a	0.476 ± 0.05 b	1.283 ± 0.16 a	0.475 ± 0.22 b	1.283 ± 0.16 a	0.451 ± 0.34 b
Anthocyanin (µg g^{-1} FW)	71.490 ± 1.9 a	55.385 ± 4.46 b	71.49 ± 1.9 a	56.956 ± 1.87 b	71.49 ± 1.9 a	48.267 ± 3.24 b
FVFM	0.7736 ±0.033 a	0.6352 ± 0.023 b	0.7736 ± 0.033 a	0.3868 ± 0.033 b	0.7736 ± 0.033 a	0.3739 ± 0.024b
qp	0.7852 ±0.0263a	0.6305 ± 0.0375a	0.785 ± 0.0263a	0.4482 ± 0.0188b	0.785 ± 0.0263a	0.3653 ± 0.02 b
NPQ	0.334 ±0.013b	0.4253 ± 0.017a	0.334 ± 0.013 b	0.6367 ± 0.0195a	0.334 ± 0.013b	0.7033 ± 0.039a

Table S5. Plant height, Shoot fresh weight, Shoot dry weight, Root dry weight, and leaf relative water content (LRWC), Leaf chlorophyll pigments, carotenoid contents, and Anthocyanin contents, Fv/Fm, non-photochemical quenching (NPQ) and photochemical quenching (qP) of PI416937 soybean cultivars grown under drought and salinity stress conditions. Data presented are means ± SD. Different letters denote significant difference at $p < 0.05$ based on the least significant difference (LSD).

**Cultivars**	**PI416937**					
Variables	Treatments					
	Control VS Drought		Control VS Salinity		Control VS drought salinity	
	Control	Drought	CK	salinity	CK	drought + salinity
plant height (cm plant-1)	69.233 ± 1.12 a	39.43 ± 1.06 b	69.233 ± 1.12 a	36.267 ± 1.20 b	69.233 ± 1.12 a	20.367 ± 0.03 b
Shoot fresh weight (mg g-1 FW)	18.100 ± 0.22 a	7.927 ± 0.30 b	18.100 ± 0.22 a	6.7733 ± 0.38 b	18.100 ± 0.22 a	4.680 ± 0.20 b
Shoot dry weight (mg g-1 FW)	8.833 ± 0.22 a	4.143 ± 0.64 b	8.833 ± 0.22 a	4.410 ± 0.46 b	8.833 ± 0.22 a	2.263 ± 0.26 b
Root dry weight (mg g-1 FW)	3.100 ± 0.11 a	1.190 ± 0.14 b	3.100 ± 0.11 a	1.243 ± 0.12 b	3.100 ± 0.11 a	0.497 ± 0.26 b
LRWC (%)	83.92 ±`3.21 a	50.5 1± 1.27`b	83.92 ±`3.21 a	60.79 ± 1.35 b	83.92 ±`3.21 a	48.47 ±`2.09 b
Chl a (mg g^{-1} FW)	1.388 ± 0.88 a	0.625 ± 0.03 b	1.388 ± 0.88 a	0.475 ± 0.01 b	1.388 ± 0.88 a	0.231 ± 0.07 b
Chl b (mg g^{-1} FW)	5.149 ± 0. 10 a	2.380 ± 0.09 b	5.149 ± 0. 10 a	2.231 ± 0.36 b	5.149 ± 0. 10 a	1.728 ± 0.17 b
Total Chl (mg g^{-1} FW)	6.537 ± 0.43 a	3.005 ± 0.64 b	6.537 ± 0.43 a	2.706 ± 0.57b	6.537 ± 0.43 a	1.959 ± 0.54 b
Carotenoid (mg g^{-1} FW)	0.944 ± 0.27a	0.2887 ± 0.18 ab	0.944 ± 0.27a	0.256 ± 0.45 b	0.944 ± 0.27a	0.174 ± 0.33 b
Anthocyanin (µg g^{-1} FW)	76.596 ± 1.12 a	57.676 ± 1.33 b	76.596 ± 1.12 a	42.815 ± 4.48 b	76.596 ± 1.12 a	32.144 ± 4.67 b
FVFM	0.7232 ± 0.012a	0.4577 ± 0.0312a	0.7232 ± 0.012a	0.236 ± 0.016 b	0.7232 ± 0.013 a	0.3739 ± 0.012 b
qp	0.7852 ± 0.0389	0.5011 ± 0.0223a	0.7852 ± 0.0389	0.4367 ± 0.010b	0.7852 ± 0.0389	0.1934 ± 0.021b
NPQ	0.3133 ± 0.017 b	0.4783 ± 0.0137 a	0.3133 ± 0.0176 b	0.7447 ±0.0131 a	0.3133 ± 0.0176 b	0.8107 ±0.0275a

Table S6. Plant height, Shoot fresh weight, Shoot dry weight, Root dry weight, and leaf relative water content (LRWC), Leaf chlorophyll pigments, carotenoid contents, and Anthocyanin contents, Fv/Fm, non-photochemical quenching (NPQ) and photochemical quenching (qP) of PI567690 soybean cultivars grown under drought and salinity stress conditions. Data presented are means ± SD. Different letters denote significant difference at $p < 0.05$ based on the least significant difference (LSD).

**Cultivars**	**PI567690**					
Variables	Treatments					
	Control VS Drought		Control VS Salinity		Control VS drought salinity	
	Control	Drought	CK	salinity	CK	drought + salinity
Plant height (cm plant-1)	66.567 ± 2.27 a	43.91± 1.02 b	66.567 ± 2.27 a	40.85 ± 2.40 b	66.567 ± 2.27 a	27.68 ± 0.76 b
Shoot fresh weight (mg g-1 FW)	18.233 ± 0.32 a	10.300 ± 0.34 b	18.233 ± 0.32 a	8.933 ± 0.40 b	18.233 ± 0.32 a	5.657 ± 0.18 b
Shoot dry weight (mg g-1 FW)	9.433 ± 0.65 a	7.633 ± 0.55 a	9.433 ± 0.65 a	5.27 ± 0.17 b	9.433 ± 0.65 a	2.867 ± 0.18 b
Root dry weight (mg g-1 FW)	3.20 ± 0.07 a	1.280 ± 0.55 b	3.20 ± 0.07 a	0.920 ± 0.18 b	3.20 ± 0.07 a	0.727 ± 0.18 b
LRWC (%)	83.119 ± 2.57 a	70.96 ± 1.89 a`	83.119 ± 2.57 a	65.831 ± 1.57 b`	83.119 ± 2.57 a	55.514 ± 2.81 b`
Chl a (mg g^{-1} FW)	1.437 ± 0.44 a	0.316 ± 0.06 b	1.437 ± 0.44 a	0.251 ± 0.08 b	1.437 ± 0.44 a	0.114 ± 0.03 b
Chl b (mg g^{-1} FW)	4.994 ± 0.14 a	2.771 ± 0.28 b	4.994 ± 0.14 a	2.883 ± 0.19 b	4.994 ± 0.14 a	1.922 ± 0.11 b
Total Chl (mg g^{-1} FW)	6.431 ± 1.13 a	3.087 ± 0.74 b	6.431 ± 1.13 a	3.134 ± 0.22 b	6.431 ± 1.13 a	2.063 ± 0.29 b
Carotenoid (mg g^{-1} FW)	1.0172 ± 1.11a	0.337 ± 0.55 b	1.0172 ± 1.11a	0.423 ± 0.13 b	1.017 ± 1.11a	0.272 ± 0.65 b
Anthocyanin (µg g^{-1} FW)	78.691 ± 2.85 a	52.544 ± 0.79 b	78.691 ± 2.85 a	51.784 ± 2.74 b	78.691 ± 2.85 a	42.750 ± 3.53 b
FVFM	0.7631 ± 0.045 a	0.5465 ± 0.0214 a	0.7631 ± 0.045 a	0.3308 ±0.0382 b	0.7631 ± 0.045 a	0.2004 ± 0.016 b
qp	0.7588 ± 0.043a	0.505 ± 0.0246a	0.7588 ± 0.043a	0.3554 ±0.0306b	0.7588 ± 0.043a	0.1637 ± 0.0336b
NPQ	0.2627 ± 0.029 a	0.5647 ± 0.0124 b	0.2627 ± 0.029 b	0.5043 ± 0.022 a	0.2627 ± 0.029 b	0.8243 ± 0.032 a

Table S7. Plant height, Shoot fresh weight, Shoot dry weight, Root dry weight, and leaf relative water content (LRWC), Leaf chlorophyll pigments, carotenoid contents, and Anthocyanin contents, Fv/Fm, non-photochemical quenching (NPQ) and photochemical quenching (qP) of PI408105A soybean cultivars grown under drought and salinity stress conditions. Data presented are means ± SD. Different letters denote significant difference at $p < 0.05$ based on the least significant difference (LSD).

**Cultivars**	**PI408105A**					
Variables	Treatments					
	Control VS Drought		Control VS Salinity		Control VS drought salinity	
	Control	Drought	CK	salinity	CK	drought + salinity
plant height (cm plant-1)	62.93 ± 2.90 a	22.03 ± 1.11 b	62.93 ± 2.90 a	25.43 ± 1.93 b	62.93 ± 2.90 a	17.33 ± 1.40 b
Shoot fresh weight (mg g-1 FW)	16.74 ± 0.90 a	5.480 ± 0.30 b	16.74 ± 0.90 a	6.28 ± 0.23 b	16.74 ± 0.90 a	4.657 ± 0.23 b
Shoot dry weight (mg g-1 FW)	8.267 ± 0.40 a	2.573 ± 0.33 b	8.267 ± 0.40 a	4.023 ± 0.86 b	8.267 ± 0.40 a	1.633 ± 0.22 b
Root dry weight (mg g-1 FW)	2.897 ± 0.08 a	0.887 ± 0.03 b	2.897 ± 0.08 a	1.030 ± 0.08 b	2.897 ± 0.08 a	0.347 ± 0.22 b
LRWC (%)	80.39 ± 4.44 a	61.78 ± 3.52 b`	80.39 ± 4.44 a	42.48 ±`2.45 b	80.39 ± 4.44 a	41.39 ± 1.53 b
Chl a (mg g^{-1} FW)	1.388 ± 0.055a	0.215± 0.03 b	1.388 ± 0.055a	0.233 ± 0.21b	1.388 ± 0.055a	0.158 ± 0.14 b
Chl b (mg g^{-1} FW)	4.902 ± 0.29 a	2.225 ± 0.15 b	4.902 ± 0.29 a	2.356 ± 0.17 b	4.902 ± 0.29 a	1.421 ± 0.12 b
Total Chl (mg g^{-1} FW)	6.29 ± 1.08 a	2.44 ± 0.98b	6.29 ± 1.08 a	2.589 ± 0.65b	6.29 ± 1.08 a	1.579 ± 0.54 b
Carotenoid (mg g^{-1} FW)	0.967 ± 0.88 a	0.181 ± 0.57 b	0.967 ± 0.88 a	0.254 ± 0.14 b	0.967 ± 0.88 a	0.1284 ± 0.44 b
Anthocyanin (µg g^{-1} FW)	80.262 ± 6.17 a	46.94 ± 3.88 b	80.262 ± 6.17 a	50.082 ± 5.57 b	80.262 ± 6.17 a	30.311 ± 0.74 b
FVFM	0.7794 ± 0.015 a	0.2997 ± 0.0328 b	0.7794 ± 0.0153 a	0.3009 ± 0.039 b	0.7794 ± 0.015 a	0.0922 ± 0.026 b
Qp	0.7588 ± 0.021a	0.3404 ± 0.0513b	0.7588 ± 0.0211a	0.2442 ± 0.036 b	0.7588 ± 0.021a	0.1179 ± 0.017 b
NPQ	0.4167 ± 0.018 b	0.6693 ± 0.0307 a	0.4167 ± 0.0181 b	0.6367 ± 0.012a	0.4167 ± 0.018 b	0.7703 ± 0.0181 a

Table S8. Proline, Free Amino acids, Soluble Sugars, Protein, Total phenols, Total flavonoids, lipid peroxidation and Electrolyte leakage, Superoxide dismutase, Peroxidase, Catalase, and Ascorbate peroxidase of PI567731 soybean cultivars grown under drought and salinity stress conditions. Data presented are means ± SD. Different letters denote significant difference at $p < 0.05$ based on the least significant difference (LSD).

**Cultivars**	**PI567731**					
Variables	Treatments					
	Control VS Drought		Control VS Salinity		Control VS drought salinity	
	Control	Drought	CK	salinity	CK	drought + salinity
Proline content (mg g^{-1} FW)	16.465 ± 0.60 b	78.975 ± 1.001a	16.465 ± 0.60b	66.892 ± 1.227a	16.465 ± 0.60b	82.14 ± 1.203a
Soluble Sugar (mg g^{-1} FW)	4.644 ± 0.1438 b	8.929 ± 0.407a	4.644 ± 0.143b	9.3769 ± 0.244a	4.644 ± 0.144b	10.77 ± 0.504a
Amino acid content (mg g^{-1} FW)	5.143 ± 0.143 b	10.458 ± 0.4074a	5.143 ± 0.143b	10.48 ± 0.244a	5.143 ± 0.143b	14.11 ± 0.704a
Protein (mg^{-1}DM)	9.933 ± 0.359 a	6.713 ± 0.3702b	9.933 ± 0.359a	4.857 ± 0.379b	9.933 ± 0.359a	4.887 ± 0.391b
Total phenol (mg g^{-1} DW)	3.698 ± 0.479 b	8.526 ± 0.466a	3.698 ± 0.479b	9.453 ± 0.439a	3.6978 ± 0.479b	12.59 ± 0.518a
Total flavonoids (mg g^{-1} DW)	3.476 ± 0.509 b	10.94 ± 0.5261a	3.476 ± 0.509b	10.989 ± 0.418a	3.4762 ± 0.509b	15.55 ± 0.7341a
MDA (mmol g^{-1} FW)	0.110 ± 0.0293a	0.269 ± 0.0137a	0.110 ± 0.0293a	0.3245 ± 0.013a	0.1105 ± 0.0293b	0.5626 ± 0.015a
Electrolytic leakage	9.888 ± 2.202 a	14.34 ± 1.9798a	9.888 ± 2.20a	13.284 ± 1.192a	9.888 ± 2.202a	28.983 ± 2.842a
SOD activity (U g^{-1} min^{-1})	44.66 ± 4.515 b	87.28 ± 5.0092a	44.66 ± 4.515b	75.403 ± 2.606a	44.66 ± 4.515b	112.98 ± 3.767a
POD activity (U g^{-1} FW min^{-1})	187.05 ± 8.35 b	404.24 ± 9.1401a	187.05 ± 8.345b	331.44 ± 9.7912a	187.05 ± 8.345b	543.69 ± 6.767a
CAT activity (U g^{-1} FW min^{-1})	26.11 ± 3.96 b	57.175 ± 1.0941a	26.11 ± 3.958b	64.228 ± 3.7305a	26.11 ± 3.958b	76.40 ± 2.26a
APX activity (U g^{-1} FW min^{-1})	64.298 ± 4.436 b	122.45 ± 5.4194a	64.298 ± 4.4365b	105 ± 0.7243a	64.298 ± 4.4365b	165.58 ± 4.777a

Table S9. Proline, Free Amino acids, Soluble Sugars, Protein, Total phenols, Total flavonoids, lipid peroxidation and Electrolyte leakage, Superoxide dismutase, Peroxidase, Catalase, and Ascorbate peroxidase of PI416937 soybean cultivars grown under drought and salinity stress conditions. Data presented are means ± SD. Different letters denote significant difference at $p < 0.05$ based on the least significant difference (LSD).

**Cultivars**		**PI416937**				
Variables	Treatments					
	Control VS Drought		Control VS Salinity		Control VS drought salinity	
	Control	Drought	CK	salinity	CK	drought + salinity
Proline content (mg g^{-1} FW)	20.81 ± 1.044b	63.892 ± 2.255a	20.81 ± 1.0441b	60.058 ± 2.4847a	20.808 ± 1.044b	66.392 ± 0.667a
Soluble Sugar (mg g^{-1} FW)	4.653 ± 0.533b	8.105 ± 0.267a	4.653 ± 0.533b	6.625 ± 0.6064a	4.653 ± 0.533b	10.154 ± 0.474a
Amino acid content (mg g^{-1} FW)	4.892 ± 0.533b	8.083 ± 0.267a	4.892 ± 0.533a	7.049 ± 0.6064a	4.892 ± 0.533a	10.269 ± 0.774a
Protein (mg^{-1}DM)	8.597 ± 0.392a	5.339 ± 0.193a	8.597 ± 0.392a	2.4409 ±0.321b	8.597 ± 0.392a	3.4314 ± 0.28a
Total phenol (mg g^{-1} DW)	4.0367 ± 0.494b	8.693 ± 0.423a	4.0367 ± 0.494b	6.86 ± 0.3043a	4.0367 ± 0.494b	12.577 ± 0.370a
Total flavonoids (mg g^{-1} DW)	3.218 ± 0.139b	10.665 ± 0.679a	3.218 ± 0.139b	8.414 ± 0.3142a	3.218 ± 0.139b	13.542 ± 0.728a
MDA (mmol g^{-1} FW)	0.182 ± 0.013b	0.268 ± 0.0234a	0.182 ± 0.0134b	0.5763 ± 0.0378a	0.1816 ± 0.013b	0.6911 ± 0.010a
Electrolytic leakage	11.23 ± 1.136a	16.121 ± 3.0643a	11.223 ± 1.136b	22.87 ± 2.1353a	11.226 ± 1.136a	30.743 ± 2.296a
SOD activity (U g^{-1} min^{-1})	47.5 ± 1.825b	80.283 ± 1.71a	47.5 ± 1.825b	69.01 ± 3.2029a	47.5 ± 1.825b	90.19 ± 3.664a
POD activity (U g^{-1} FW min^{-1})	168.39 ± 6.120b	353.89 ± 7.522a	168.39 ± 6.120b	258.64 ± 5.7965a	168.39 ± 6.120b	502.14 ± 11.541a
CAT activity (U g^{-1} FW min^{-1})	22.38 ± 1.326b	52.293 ± 4.396a	22.38 ± 1.326b	52.171 ± 4.1543a	22.379 ± 1.326b	68.502 ± 2.715a
APX activity (U g^{-1} FW min^{-1})	48.55 ± 5.328b	103.87 ± 3.921a	48.55 ± 5.328b	77.33 ±3.124a	48.547 ± 5.328b	145.04 ± 4.792a

Table S10. Proline, Free Amino acids, Soluble Sugars, Protein, Total phenols, Total flavonoids, lipid peroxidation and Electrolyte leakage, Superoxide dismutase, Peroxidase, Catalase, and Ascorbate peroxidase of PI567690 soybean cultivars grown under drought and salinity stress conditions. Data presented are means ± SD. Different letters denote significant difference at $p < 0.05$ based on the least significant difference (LSD).

**Cultivars**		**PI567690**				
Variables	Treatments					
	Control VS Drought		Control VS Salinity		Control VS drought salinity	
	Control	Drought	CK	salinity	CK	drought + salinity
Proline content (mg g^{-1} FW)	21.242 ±3.34b	73.892 ±2.086a	21.242 ±3.34b	54.558 ±2.504a	21.242 ±3.339b	75.308 ±3.812a
Soluble Sugar (mg g^{-1} FW)	4.2436 ±0.345b	9.429 ±0.244a	4.2436 ±0.345b	8.2464 ±0.505a	4.2436 ±0.345b	8.542 ±0.308a
Amino acid content (mg g^{-1} FW)	5.5676 ±0.345a	8.838 ±0.244a	5.5676 ±0.345a	7.753 ±0.505a	5.5676 ±0.345b	11.841 ±0.308a
Protein (mg^{-1}DM)	10.335 ±0.599a	4.8546 ±0.046b	10.335 ±0.599a	4.400 ± 0.389a	10.335 ±0.599a	3.1359 ±0.317b
Total phenol (mg g^{-1} DW)	3.6053 ±0.372a	8.8356 ±0.519b	3.6053 ±0.372a	7.8338 ±0.380a	3.6053 ±0.372a	11.433 ±0.218b
Total flavonoids (mg g^{-1} DW)	4.4484 ±0.465b	10.983 ±0.649a	4.4484 ±0.465a	9.733 ±0.248a	4.4484 ±0.465b	14.23 ±0.522a
MDA (mmol g^{-1} FW)	0.1108 ±0.038b	0.319 ±0.035a	0.1108 ±0.038b	0.4315 ±0.016a	0.1108± 0.038b	0.6424 ±0.018a
Electrolytic leakage	9.33 ± 1.925a	16.066 ±0.595a	9.33 ±1.925a	14.099 ±1.009a	9.33 ±1.925b	32.686 ±1.378a
SOD activity (U g^{-1} min^{-1})	50.107 ±5.065a	80.047 ±2.379a	50.107 ±5.065b	70.837 ±2.436a	50.107 ±5.065b	98.92 ±1.513a
POD activity (U g^{-1} FW min^{-1})	198.64 ±7.257b	372.07 ±5.738a	198.64 ±7.257b	269.23 ±6.424a	198.64 ±7.257b	518.1 ±4.454a
CAT activity (U g^{-1} FW min^{-1})	28.696 ±3.790b	50.481 ±2.373a	28.696 ±3.790b	44.309 ±3.505a	28.696 ±3.790a	63.537 ±4.98a
APX activity (U g^{-1} FW min^{-1})	47.454 ±3.624a	97.13 ±8.702a	47.454 ±3.625b	87.77 ± 0.757a	47.454 ±3.624b	142.58 ±3.683a

Table S11. Proline, Free Amino acids, Soluble Sugars, Protein, Total phenols, Total flavonoids, lipid peroxidation and Electrolyte leakage, Superoxide dismutase, Peroxidase, Catalase, and Ascorbate peroxidase of PI408105A soybean cultivars grown under drought and salinity stress conditions. Data presented are means ± SD. Different letters denote significant difference at $p < 0.05$ based on the least significant difference (LSD).

**Cultivars**	**PI408105A**					
Variables	Treatments					
	Control VS Drought		Control VS Salinity		Control VS drought salinity	
	Control	Drought	CK	salinity	CK	drought + salinity
Proline content (mg g^{-1} FW)	22.642 ±0.542a	30.725 ±1.607a	22.642 ±0.542a	35.642 ±1.333b	22.642 ±0.542a	51.725 ±2.6827b
Soluble Sugar (mg g^{-1} FW)	4.9075 ±0.055b	7.0857 ±0.339a	4.9075 ±0.055b	7.4806 ±0.397a	4.9075± 0.055b	8.153 ±0.3083a
Amino acid content (mg g^{-1} FW)	5.064 ±0.338b	8.9953 ±0.055a	5.064 ±0.338b	8.697 ±0.398a	5.064 ±0.338a	8.011± 0.2083a
Protein (mg^{-1}DM)	7.379 ±0.121a	4.4835 ±0.565a	7.379 ±0.121a	5.4645 ±0.561a	7.379 ±0.121a	1.5946 ±0.1509b
Total phenol (mg g^{-1} DW)	3.5433 ±0.434b	6.5587 ±0.408a	3.5433 ±0.434b	8.2571 ±0.455a	3.5433 ±0.434b	9.421 ±0.3251a
Total flavonoids (mg g^{-1} DW)	4.369 ±0.542b	8.139 ±0.585a	4.369 ±0.542b	10.262 ±0.569a	4.369 ±0.542b	11.717 ±0.4063a
MDA (mmol g^{-1} FW)	0.203 ±0.021b	0.574 ±0.055a	0.2029 ±0.021b	0.6648 ±0.018a	0.2029 ±0.021b	0.8705 ±0.0234a
Electrolytic leakage	11.268 ±0.785a	25.754 ±0.801a	11.268 ±0.785a	18.008 ±0.686a	11.268 ±0.785b	42.127 ±0.8011a
SOD activity (U g^{-1} min^{-1})	40.297 ±2.120a	65.137 ±3.276a	40.297 ±2.120a	67.467 ±1.568a	40.297 ±2.120b	83.99 ±2.8378a
POD activity (U g^{-1} FW min^{-1})	232.04 ±6.53b	316 ± 7.319a	232.04 ±6.526b	335.34 ±4.3712a	232.04 ±6.526b	512.72 ±6.8382a
CAT activity (U g^{-1} FW min^{-1})	22.897 ±4.834b	48.4147 ±4.013a	22.897 ±4.8339a	52.1709 ±2.6146a	22.897 ±4.834b	56.674 ±3.9635a
APX activity (U g^{-1} FW min^{-1})	51.441± 2.718b	79.73 ±5.287a	51.441 ±2.718b	93.34 ±2.4876a	51.441 ±2.718b	149.72 ±7.2735a
